# Variational Supertrees for Bayesian Phylogenetics

**DOI:** 10.1007/s11538-024-01338-5

**Published:** 2024-08-05

**Authors:** Michael D. Karcher, Cheng Zhang, Frederic A. Matsen

**Affiliations:** 1 Department of Math & CS, Muhlenberg College, 2400 W Chew St, Allentown, PA 18104 USA; 2 School of Mathematical Sciences and Center for Statistical Science, Peking University, No. 5 Yiheyuan Road, Haidian District, Beijing, 100871 People’s Republic of China; 3 Computational Biology Program, Fred Hutchinson Cancer Research Center, 1100 Fairview Ave. N., Seattle, WA 98109 USA

**Keywords:** Supertrees, Variational methods, Phylogenetics, Gradient descent, Divide-and-conquer, 49-04, 49N99, 62-08, 92-08, 92D20, 92D30

## Abstract

Bayesian phylogenetic inference is powerful but computationally intensive. Researchers may find themselves with two phylogenetic posteriors on overlapping data sets and may wish to approximate a combined result without having to re-run potentially expensive Markov chains on the combined data set. This raises the question: given overlapping subsets of a set of taxa (e.g. species or virus samples), and given posterior distributions on phylogenetic tree topologies for each of these taxon sets, how can we optimize a probability distribution on phylogenetic tree topologies for the entire taxon set? In this paper we develop a variational approach to this problem and demonstrate its effectiveness. Specifically, we develop an algorithm to find a suitable support of the variational tree topology distribution on the entire taxon set, as well as a gradient-descent algorithm to minimize the divergence from the restrictions of the variational distribution to each of the given per-subset probability distributions, in an effort to approximate the posterior distribution on the entire taxon set.

## Introduction

Fields such as phylogenetics often work with a sort of abstracted family tree, called a *phylogenetic tree*, frequently abbreviated here as *tree*. These trees have different members of a population as their tips, and their branching points describe the relations between the tips and how recently they had a common ancestor. If some of the tips are censored, the tree topology simplifies in a process we refer to as *restriction*. If one has multiple trees restricted from the same original, uncensored tree, one may wish to reconstruct the original *supertree*. Suppose instead one has multiple probability distributions of restricted trees, then one may be interested in reconstructing the supertree probability distribution. This is a difficult problem both theoretically and computationally without additional structure. We take a variational approach by training a flexible model to approximate the true supertree distribution as closely as possible, while still maintaining computational tractability.

This problem falls in the domain of *supertree analysis*, a topic that has gone by this name since 1986 but has much earlier roots as reviewed by Sanderson et al. (1998) and Bininda-Emonds (2004). Broadly speaking, there are two goals of supertree analysis. The first goal is to reduce computational complexity by dividing the ensemble of taxa into subsets, performing independent analysis on those subsets, and then combining these analyses into a single tree (Huson et al. 1999), or in the Bayesian case a single posterior distribution. The second goal is to combine information from multiple sources, such as different genes, which may have divergent phylogenetic signal and patterns of presence and absence. Although there is some overlap between these goals, the focus of this paper is on the first goal. Algorithms for the second goal are better served by methods that explicitly model the origins of different phylogenetic signal, such as via the multispecies coalescent (Liu and Pearl 2007; Heled and Drummond 2010).

The eventual goal of our work is to provide a divide-and-conquer strategy for Bayesian phylogenetics, in which taxa are divided into subsets, a Bayesian analysis is run on each, and then knitted back together using a supertree approach. Although this approach in the non-Bayesian case has been a consistent theme in phylogenetics since the work of Huson et al. (1999), the equivalent idea in Bayesian phylogenetics is comparatively underdeveloped. This seems surprising given that Bayesian analyses are much more computationally demanding than their non-Bayesian counterparts, such that the lack of rapid Bayesian inference techniques is limiting their application in important realms such as genomic epidemiology. To motivate our method, we consider the case where researchers have already performed an expensive Bayesian phylogenetic analysis and have subsequently acquired more sequence data, as often happens in epidemiology. Our approach provides a method for combining a new, smaller phylogenetic analysis (including the new sequences) with the earlier results, reducing the need for a new, expensive analysis on all of the sequences at once.

The most relevant existing work, by Ronquist et al. (2004), summarizes phylogenetic posterior distributions in terms of one of two schemes. In the Weighted Independent Binary (WIB) scheme, a tree’s probability is proportional to a product of terms, each term being present in the product of the corresponding bipartition present in the tree. This scheme is in a sense a simpler version of the strategy presented here. The Weighted Additive Binary (WAB) scheme is an extension of the long-standing tradition in supertree analysis of performing parsimony analysis on a data matrix formed from encoding the splits of the tree as binary characters. The weighting in WAB comes from assigning weights to the characters in such an encoding according to their confidences. One can then translate the corresponding parsimony objective into a Bayesian setting by assigning a log-likelihood penalty to each unit of parsimony cost. In total, by taking posterior distributions for trees on each of the subsets, summarizing them in terms of one of these schemes, and then using products of these factors as an approximation for posterior probability. Ronquist et al. (2004) show some correlation of this method with actual tree posterior probabilities for example data sets on six and ten taxa, and that the WAB scheme outperforms the WIB scheme.

In this paper we develop a variational formulation for supertree estimation. Given a collection of reference distributions of tree topologies with overlapping tips (typically acquired via Bayesian phylogenetic inference), in order to approximate the posterior on the full tip set we find a *supertree distribution* that closely approximates each reference distribution when only considering the tips in that reference. We structure our supertree distribution using subsplit Bayesian networks (*SBNs*) (Zhang and Matsen IV 2018) reviewed below, which generalize previous formalisms for describing probability distributions on topologies (Höhna and Drummond 2012; Larget 2013). We note in passing that these formalisms, in turn, noted connections between their methods and the supertree work of Ronquist et al. (2004). We focus on the case where the reference distributions are originally given as, or subsequently approximated by, SBNs, but the method is generalizable to arbitrary reference distributions at the cost of computational efficiency. We accomplish our goal of training a supertree distribution using gradient descent to minimize the differences between our reference distributions and our supertree distribution (appropriately restricted). Moreover, we show that the method successfully trains a supertree distribution that is close to the original posterior (SBN) on both simulated and real-world phylogenetic sequence data.

## Methods

### Overview

**Fig. 1:**
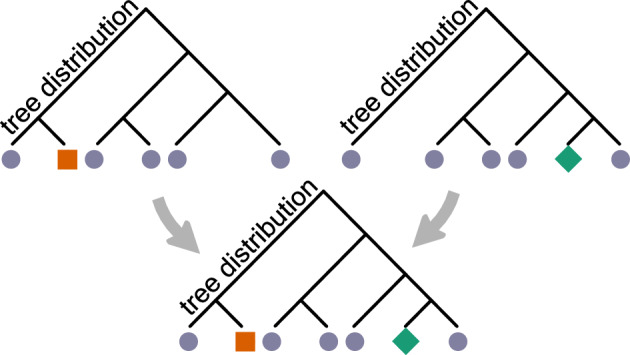
Illustrating a supertree method on distributions. The top row of two tree distributions are reference distributions, and the bottom tree distribution is the supertree distribution. The circles indicate tips shared by the two reference distributions, whereas the square and diamond represent tips that are only present in one of the two references

Suppose we are given a set of probability distributions $\{p_i\}$ on rooted, bifurcating phylogenetic tree topologies, abbreviated as *tree topologies* or simply *topologies*, each with a corresponding tip set $X_i$. We refer to these *tree distributions* on their respective tip sets as our *reference distributions*. These reference distributions should be thought of as the input data for this method. We construct a supertree distribution by finding a probability distribution $q(\tau )$ of topologies on the entire taxon set $X = \cup _i X_i$ so that *q* is as close as possible to each of the reference distributions when the tips not present in that reference distribution are removed. Figure 1 illustrates the flow of information, taking two reference distributions and producing a supertree distribution on the union of its references’ tip sets.

**Fig. 2:**
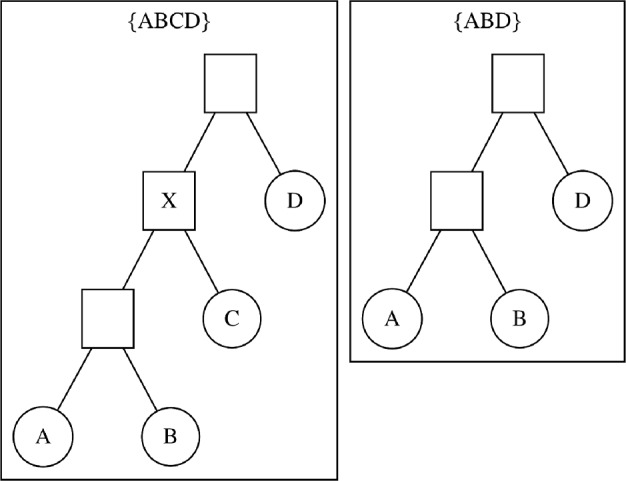
Illustration of restricting tree topologies. We restrict a tree $\tau$ with tip set $\{ABCD\}$ (left) to tip set $\{ABD\}$, resulting in the tree $\tau \mathop {\hspace{-1.66656pt}\downharpoonright \hspace{-1.111pt}\mathchoice{\hspace{-1.94443pt}}{\hspace{-1.94443pt}}{}{}_{{ABD}}} \mathchoice{\hspace{-0.55542pt}}{\hspace{-0.55542pt}}{\hspace{-0.27771pt}}{}$ (right). We remove tip *C* and the internal node marked with an *X* as it no longer has two descendants

We now establish the formalisms necessary to achieve this goal. Given a taxon subset ${\bar{X}} \subset X$ and a topology $\tau$ on *X*, define the *restriction* $\tau \mathop {\hspace{-1.66656pt}\downharpoonright \hspace{-1.111pt}\mathchoice{\hspace{-1.94443pt}}{\hspace{-1.94443pt}}{}{}_{\bar{X}}} \mathchoice{\hspace{-0.55542pt}}{\hspace{-0.55542pt}}{\hspace{-0.27771pt}}{}$ to be the topology induced by removing all taxa that are not in ${\bar{X}}$ from $\tau$ and removing all internal nodes with fewer than two children (Semple and Steel 2003). We illustrate an example of this process in Fig. 2. Given a probability distribution *q* on tree topologies with taxon set *X* and a topology ${\bar{\tau }}$ on ${\bar{X}}$, we define the restriction of *q* to ${\bar{X}}$ as a marginalization over the topologies that restrict to ${\bar{\tau }}$,

1 $$\begin{aligned} q \mathop {\hspace{-1.66656pt}\downharpoonright \hspace{-1.111pt}\mathchoice{\hspace{-1.94443pt}}{\hspace{-1.94443pt}}{}{}_{\bar{X}}} \mathchoice{\hspace{-0.55542pt}}{\hspace{-0.55542pt}}{\hspace{-0.27771pt}}{} ({{\bar{\tau }}}) :=\sum _{\tau : \tau \mathop {\hspace{-1.66656pt}\downharpoonright \hspace{-1.111pt}\mathchoice{\hspace{-1.94443pt}}{\hspace{-1.94443pt}}{}{}_{\bar{X}}} \mathchoice{\hspace{-0.55542pt}}{\hspace{-0.55542pt}}{\hspace{-0.27771pt}}{} = {\bar{\tau }}} q(\tau ). \end{aligned}$$

In this paper, our goal is to infer a distribution $q(\tau )$ for topologies on the entire taxon set *X* such that its restrictions $\{ q \mathop {\hspace{-1.66656pt}\downharpoonright \hspace{-1.111pt}\mathchoice{\hspace{-1.94443pt}}{\hspace{-1.94443pt}}{}{}_{X_i}} \mathchoice{\hspace{-0.55542pt}}{\hspace{-0.55542pt}}{\hspace{-0.27771pt}}{} \}$ resemble the corresponding reference distributions $\{p_i\}$. In the use case where the reference distributions are all samples from restrictions of one phylogenetic tree posterior on the full tip set, our ultimate goal is to approximate the full posterior using the supertree distribution. Mechanically, our objective will be to minimize our *loss function* *L*: the sum of KL-divergences between each reference distribution and *q* restricted to its taxon subset,

$$\begin{aligned} L(\{p_i\} \parallel q) = \sum _i D_{\text {KL}}(p_i \parallel q \mathop {\hspace{-1.66656pt}\downharpoonright \hspace{-1.111pt}\mathchoice{\hspace{-1.94443pt}}{\hspace{-1.94443pt}}{}{}_{X_i}} \mathchoice{\hspace{-0.55542pt}}{\hspace{-0.55542pt}}{\hspace{-0.27771pt}}{} ) = \sum _i \left[ -\sum _{\tau } p_i(\tau ) \log \left( \frac{ q \mathop {\hspace{-1.66656pt}\downharpoonright \hspace{-1.111pt}\mathchoice{\hspace{-1.94443pt}}{\hspace{-1.94443pt}}{}{}_{X_i}} \mathchoice{\hspace{-0.55542pt}}{\hspace{-0.55542pt}}{\hspace{-0.27771pt}}{} (\tau )}{p_i(\tau )} \right) \right] . \end{aligned}$$

Also note that the KL-divergence will be undefined for any tree $\tau$ where $q \mathop {\hspace{-1.66656pt}\downharpoonright \hspace{-1.111pt}\mathchoice{\hspace{-1.94443pt}}{\hspace{-1.94443pt}}{}{}_{X_i}} \mathchoice{\hspace{-0.55542pt}}{\hspace{-0.55542pt}}{\hspace{-0.27771pt}}{} (\tau )=0$, so some care must be taken to ensure support compatibility.

If we have reason to prioritize some reference distributions differently than others, due to differing confidence in the different distributions among other reasons, we can easily incorporate weights into a weighted loss function,

$$\begin{aligned} L(\{p_i, w_i\} \parallel q) = \sum _i w_i D_{\text {KL}}(p_i \parallel q \mathop {\hspace{-1.66656pt}\downharpoonright \hspace{-1.111pt}\mathchoice{\hspace{-1.94443pt}}{\hspace{-1.94443pt}}{}{}_{X_i}} \mathchoice{\hspace{-0.55542pt}}{\hspace{-0.55542pt}}{\hspace{-0.27771pt}}{} ).\end{aligned}$$

For the algorithms that follow in this paper, any choice of positive weights would be appropriate. However, hereafter we focus on the unweighted version.

For parameterizations of *q* such that $D_{\text {KL}}(p_i \parallel q \mathop {\hspace{-1.66656pt}\downharpoonright \hspace{-1.111pt}\mathchoice{\hspace{-1.94443pt}}{\hspace{-1.94443pt}}{}{}_{X_i}} \mathchoice{\hspace{-0.55542pt}}{\hspace{-0.55542pt}}{\hspace{-0.27771pt}}{} )$ has an efficiently computable gradient with respect to *q*’s parameters, gradient descent is available for minimizing the loss function. We describe one such family of parameterizations using SBNs below and derive efficient KL-divergences and gradients later in this section.

### Review of Subsplit Bayesian Networks

Here we review subsplit Bayesian networks (SBNs) in the case of rooted topologies. Our approach will have a different emphasis than the original Zhang and Matsen IV (2018) work. Where the previous work described SBNs as a very general class of Bayesian networks and concentrated on unrooted trees, we will focus on a simpler SBN structure parameterizing rooted, bifurcating (phylogenetic) trees.

We will use the term *clade* to refer to a subset of a taxon set *X*. A *subsplit* is a set containing two disjoint *child clades* $s = \{Y, Z\}$. We define the *parent clade* of a subsplit as the union of its two child clades, with notation $U(s) = Y \cup Z$. If we need to specify a particular child clade *Y* of a subsplit $s = \{Y, Z\}$ as being the focus of attention (as opposed to the other child clade), we use the notations $({s},\underline{Y})$ or $\{Z, \underline{Y}\}$. Note that the parent clade of *s* is allowed to be a proper subset of *X*, in contrast to the traditional definition of a *split* as a bipartition of the entire taxon set *X* (Semple and Steel 2003). We will say that the subsplit *s* *divides* a clade *W* if $U(s) = W$, with notation $W \rightarrow s$. Note that due to our bifurcating assumption, every clade of size two or larger will be divided by a subsplit. We also say that *s* is a *child subsplit* of *parent subsplit* *t* if *U*(*s*) is a child clade of *t*. We refer to *t* and *s* as a *parent–child subsplit pair* or *PCSP* with notation $t \rightarrow s$ when *s* is a valid child of a subsplit *t*.

We also extend the concept of valid child subsplits to further descendants. Given subsplit $a = \{Y, Z\}$, we say that subsplit *d* is a valid *descendant* of $({a},\underline{Y})$ if $U(d) \subseteq Y$, and we use the notation $({a},\underline{Y}) \rightarrow _*d$. Additionally, we say that *d* is a valid descendant of *a* with notation $a \rightarrow _*d$ if $({a},\underline{Y}) \rightarrow _*d$, $({a},\underline{Z}) \rightarrow _*d$, or $a = d$. Equivalently, we say *a* is a valid *ancestor* of *d* under the same conditions and with the same notation. Note that $t \rightarrow s$ implies $t \rightarrow _*s$ but not vice versa.

We use the term *path* to refer to a sequence of subsplits such that each element is a descendant of the previous. For example, the path $a \rightarrow _*t \rightarrow s$ would refer to a sequence starting with *a*, proceeding via any number of subsplits to *t* (including zero if $a=t$), then directly to *t*’s child *s*.

It will be convenient to also introduce *singletons* and *trivial subsplits*. A singleton corresponds to one of the tips of the tree and is represented by a clade with size one or a subsplit containing a singleton clade and the empty set. A subsplit is trivial if one of its child clades is empty. We typically exclude singletons and trivial subsplits from sets of subsplits, unless explicitly included.

Each bifurcating rooted topology can be uniquely represented as a set of the subsplits it contains. For example, the topology given by the Newick string (Felsenstein 1986) "((t1,t2),t3);" is described by the subsplits $\{\{\texttt{t1},\texttt{t2}\}, \{\texttt{t3}\}\}$ and $\{\{\texttt{t1}\}, \{\texttt{t2}\}\}$. We will use the notation $s \in \tau$ to mean that the subsplit *s* is found in $\tau$ and the notation $\tau \subseteq S$ to mean that all of the subsplits in $\tau$ are in set *S*. The same holds true for specifying a topology in terms of PCSPs, and we will use the same notation in that case. Each subsplit $s \in \tau$ has two child clades which each must correspond to a singleton or a subsplit that divides it. Similarly, for a given topology $\tau$ each tip or subsplit *s* has a *parent subsplit* *t* such that *U*(*s*) is one of the child clades of *t*. We will denote the parent subsplit of *s* with $\pi _{\tau }{(s)}$. In the above example, $\{\{\texttt{t1},\texttt{t2}\}, \{\texttt{t3}\}\}$ is the parent of $\{\{\texttt{t1}\}, \{\texttt{t2}\}\}$, which in turn is the parent of singletons $\{\texttt{t1}\}$ and $\{\texttt{t2}\}$. In order to eliminate having to make a special case for the root subsplit *r*, we define its parent subsplit to be a special trivial subsplit of the entire taxon set, i.e. $\pi _{\tau }{(r)} = \{X, \emptyset \} = \pi _{X}$.

In order to illustrate how to construct an SBN, we first describe how to sample a topology from an SBN. Starting from the root clade, recursively construct a topology: for any currently-childless clade *W* larger than a singleton, sample a subsplit that divides *W* from a probability distribution, supplied by the SBN, conditional on some subset of the ancestors of *W*. These conditional distributions can be parameterized in different ways using different subsets of the clades’ ancestry (Zhang and Matsen IV 2018), with each parameterization defining a family of SBN probability distributions on tree topologies. In this paper, we focus on two families in particular: *clade-conditional distributions* (CCDs) where the subsplit distributions $p(s \vert U(s))$ are conditional on the subsplits’ parent clade (Höhna and Drummond 2012; Larget 2013), and *subsplit-conditional distributions* (SCDs) where the subsplit distributions $p(s \vert ({t},\underline{U(s)}))$ are conditional on the subsplits’ parent subsplit and clade (Zhang and Matsen IV 2018). We choose these two parameterizations for different reasons: CCDs for simplicity and elegance, illustrating the supertree algorithms, and SCDs because they can be trained to a higher fidelity to a target distribution (Zhang and Matsen IV 2018).

We fix the conditional probability of any singleton to be 1, and with our induced conditional independence assumptions, the SBN probability for a rooted tree $\tau$ can then be easily computed:

- under CCDs $$p(\tau ) = \prod _{s \in \tau } p(s \vert U(s))$$,
- under SCDs $$p(\tau ) = \prod _{s \in \tau } p(s \vert ({\pi _{\tau }{(s)}},\underline{U(s)}))$$.

We use the notation $p(s) :=p(s \in \tau )$ for the *unconditional* probability of a subsplit *s* being present in a topology $\tau$ randomly sampled according to *p*. Similarly, we use $p(t \rightarrow s)$ for the unconditional probability of PCSP $t \rightarrow s$, namely $p(s \vert ({t},\underline{U(s)})) \, p(t)$. For CCD-parameterized SBN *p*, we define the *subsplit support* ${\mathscr {C}}_{}$ as the set of subsplits that have positive probability under *p*. For SCD-parameterized SBN *p*, we define the *PCSP support* ${\mathscr {P}}_{}$ as a heterogeneous set containing the PCSPs that have positive probability under *p*, the subsplits that have positive probability under *p*, the singletons for *p*’s tip set, and the empty subsplit.

### KL-Divergence Between SBNs

Here we show that the KL-divergence between two SBN-parameterized distributions can be computed efficiently. If both $p(\tau )$ and $q(\tau )$ are CCD-parameterized SBNs,

2 $$\begin{aligned} D_{\text {KL}}(p \parallel q)&= -\sum _{\tau } p(\tau ) \log \left( \frac{q(\tau )}{p(\tau )} \right) \nonumber \\&= -\sum _{\tau } p(\tau ) \sum _{s} 1_{s \in \tau } \log \left( \frac{q(s \vert U(s))}{p(s \vert U(s))} \right) \nonumber \\&= -\sum _{s} \log \left( \frac{q(s \vert U(s))}{p(s \vert U(s))} \right) \sum _{\tau } p(\tau ) 1_{s \in \tau } \nonumber \\&= -\sum _{s} p(s) \, \left[ \log \left( q(s \vert U(s)) \right) - \log \left( p(s \vert U(s)) \right) \right] . \end{aligned}$$

Computing this sum is linear time in the number of subsplits in the subsplit support of *p*.

Similarly, if both $p(\tau )$ and $q(\tau )$ are SCD-parameterized SBNs,

3 $$\begin{aligned} D_{\text {KL}}(p \parallel q)&= -\sum _{\tau } p(\tau ) \log \left( \frac{q(\tau )}{p(\tau )} \right) \nonumber \\&= -\sum _{\tau } p(\tau ) \sum _{(t \rightarrow s)} 1_{(t \rightarrow s) \in \tau } \log \left( \frac{q(s \vert ({t},\underline{U(s)}))}{p(s \vert ({t},\underline{U(s)}))} \right) \nonumber \\&= -\sum _{(t \rightarrow s)} \log \left( \frac{q(s \vert ({t},\underline{U(s)}))}{p(s \vert ({t},\underline{U(s)}))} \right) \sum _{\tau } p(\tau ) 1_{(t \rightarrow s) \in \tau } \nonumber \\&= -\sum _{(t \rightarrow s)} p(t \rightarrow s) \, \left[ \log \left( q(s \vert ({t},\underline{U(s)})) \right) - \log \left( p(s \vert ({t},\underline{U(s)})) \right) \right] . \end{aligned}$$

Computing this sum is linear time in the number of PCSPs in the PCSP support of *p*.

### Restricting SBNs

Equation 1 defines how to take a distribution *q* on trees with taxon set *X* and restrict it to its induced distribution on trees with taxon set $\bar{X} \subset X$. If *q* is an SBN-parameterized distribution, we can more efficiently calculate $q \mathop {\hspace{-1.66656pt}\downharpoonright \hspace{-1.111pt}\mathchoice{\hspace{-1.94443pt}}{\hspace{-1.94443pt}}{}{}_{\bar{X}}} \mathchoice{\hspace{-0.55542pt}}{\hspace{-0.55542pt}}{\hspace{-0.27771pt}}{}$ from the SBN parameters directly: we can restrict a subsplit *s* to taxon set $\bar{X}$ by taking the intersection of both child clades with $\bar{X}$. One consequence of this is that some subsplits on *X* will become trivial subsplits on $\bar{X}$. On the other hand, if a restricted subsplit is nontrivial, then we know the original subsplit is nontrivial, because the restricted subsplit separates at least one pair of tips, so the original subsplit will separate those tips at well. Furthermore, subsplits represent recursive bipartitions of sets, so any pair of tips can only be partitioned by a subsplit once. Therefore, no two subsplits that restrict to the same nontrivial subsplit $\bar{s}$ can appear in the same tree, since any subsplit that restricts to $\bar{s}$ separates all the same tips that $\bar{s}$ partitions. By this mutual exclusivity, the probability of a restricted subsplit $\bar{s}$ under a restricted distribution $q \mathop {\hspace{-1.66656pt}\downharpoonright \hspace{-1.111pt}\mathchoice{\hspace{-1.94443pt}}{\hspace{-1.94443pt}}{}{}_{\bar{X}}} \mathchoice{\hspace{-0.55542pt}}{\hspace{-0.55542pt}}{\hspace{-0.27771pt}}{}$ is simply

4 $$\begin{aligned} q \mathop {\hspace{-1.66656pt}\downharpoonright \hspace{-1.111pt}\mathchoice{\hspace{-1.94443pt}}{\hspace{-1.94443pt}}{}{}_{\bar{X}}} \mathchoice{\hspace{-0.55542pt}}{\hspace{-0.55542pt}}{\hspace{-0.27771pt}}{} (\bar{s}) = \sum _{s: \, s \mathop {\hspace{-1.66656pt}\downharpoonright \hspace{-1.111pt}\mathchoice{\hspace{-1.94443pt}}{\hspace{-1.94443pt}}{}{}_{\bar{X}}} \mathchoice{\hspace{-0.55542pt}}{\hspace{-0.55542pt}}{\hspace{-0.27771pt}}{} = \bar{s}} q(s). \end{aligned}$$

Similarly, subsplits with the same clade are mutually exclusive, so the unconditional probability of a clade appearing is

5 $$\begin{aligned} q \mathop {\hspace{-1.66656pt}\downharpoonright \hspace{-1.111pt}\mathchoice{\hspace{-1.94443pt}}{\hspace{-1.94443pt}}{}{}_{\bar{X}}} \mathchoice{\hspace{-0.55542pt}}{\hspace{-0.55542pt}}{\hspace{-0.27771pt}}{} (\bar{U}) = \sum _{\bar{s}': \, U(\bar{s}')=\bar{U}} q \mathop {\hspace{-1.66656pt}\downharpoonright \hspace{-1.111pt}\mathchoice{\hspace{-1.94443pt}}{\hspace{-1.94443pt}}{}{}_{\bar{X}}} \mathchoice{\hspace{-0.55542pt}}{\hspace{-0.55542pt}}{\hspace{-0.27771pt}}{} (\bar{s}'). \end{aligned}$$

In order to construct the restricted SBN, we need to compute the appropriate conditional probabilities, which we can easily calculate from unconditional probabilities. In a CCD context, subsplit *s* probabilities are conditional on observing its clade *U*(*s*). We can build upon Eq. 1 to find the restricted SBN induced by restricting to $\bar{X} \subset X$. We see

6 $$\begin{aligned} q \mathop {\hspace{-1.66656pt}\downharpoonright \hspace{-1.111pt}\mathchoice{\hspace{-1.94443pt}}{\hspace{-1.94443pt}}{}{}_{\bar{X}}} \mathchoice{\hspace{-0.55542pt}}{\hspace{-0.55542pt}}{\hspace{-0.27771pt}}{} (\bar{s} \mid U(\bar{s})) = \frac{ q \mathop {\hspace{-1.66656pt}\downharpoonright \hspace{-1.111pt}\mathchoice{\hspace{-1.94443pt}}{\hspace{-1.94443pt}}{}{}_{\bar{X}}} \mathchoice{\hspace{-0.55542pt}}{\hspace{-0.55542pt}}{\hspace{-0.27771pt}}{} (U(\bar{s}) \rightarrow \bar{s})}{ q \mathop {\hspace{-1.66656pt}\downharpoonright \hspace{-1.111pt}\mathchoice{\hspace{-1.94443pt}}{\hspace{-1.94443pt}}{}{}_{\bar{X}}} \mathchoice{\hspace{-0.55542pt}}{\hspace{-0.55542pt}}{\hspace{-0.27771pt}}{} (U(\bar{s}))} = \frac{ q \mathop {\hspace{-1.66656pt}\downharpoonright \hspace{-1.111pt}\mathchoice{\hspace{-1.94443pt}}{\hspace{-1.94443pt}}{}{}_{\bar{X}}} \mathchoice{\hspace{-0.55542pt}}{\hspace{-0.55542pt}}{\hspace{-0.27771pt}}{} (\bar{s})}{ q \mathop {\hspace{-1.66656pt}\downharpoonright \hspace{-1.111pt}\mathchoice{\hspace{-1.94443pt}}{\hspace{-1.94443pt}}{}{}_{\bar{X}}} \mathchoice{\hspace{-0.55542pt}}{\hspace{-0.55542pt}}{\hspace{-0.27771pt}}{} (U(\bar{s}))}. \end{aligned}$$

**Fig. 3:**
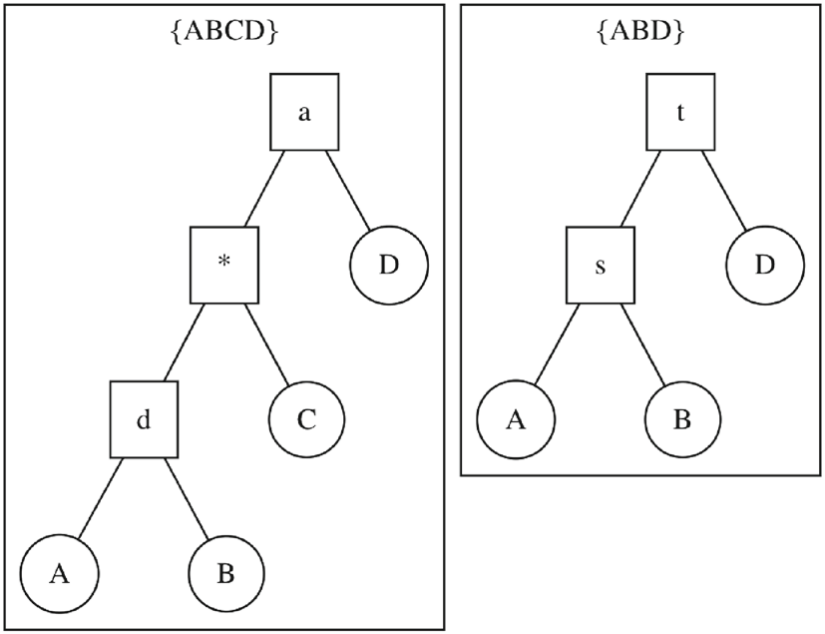
Illustration of restricting PCSPs. We restrict a tree with tip set $\{ABCD\}$ (left) to tip set $\{ABD\}$ (right). Note how the node with label *a* on the left corresponds to the node with label *t* on the right, just as the node with label *d* corresponds to the node with label *s*. Note that nodes *a* and *d* are not parent and child, but nodes *t* and *s* are. This is possible because the node with label $*$ is trivial under restriction, and therefore has no corresponding node on the right

A slightly more involved construction is needed to gain an equivalent formula for the SCD case. For SCD parameterizations, we need the unconditional probability of a PCSP. PCSPs are mutually exclusive, so an argument similar to the above holds, but subsplits *a* and *d* that respectively restrict to the restricted parent $\bar{t}$ and child $\bar{s}$ do not themselves have to be a valid parent–child pair before restriction. We illustrate this possibility in Fig. 3. More formally, the following two statements are equivalent: (1) the ancestor–descendant pair $a \rightarrow _*d$ restricts to the PCSP $\bar{t} \rightarrow \bar{s}$, and (2) a sequence of parent–child pairs in *q* exists, starting with *a*, ending with *d*, with subsplits $\{t_i\}$ in between, such that each $t_i \mathop {\hspace{-1.66656pt}\downharpoonright \hspace{-1.111pt}\mathchoice{\hspace{-1.94443pt}}{\hspace{-1.94443pt}}{}{}_{\bar{X}}} \mathchoice{\hspace{-0.55542pt}}{\hspace{-0.55542pt}}{\hspace{-0.27771pt}}{}$ is trivial. The converse is elementary, but to show that (1) implies (2), we know *a* and *d* exist by assumption, and *a* restricts to $\bar{t}$ which is the parent of $\bar{s}$. Then one of *a*’s child clades restricts to $U(\bar{s})$, and every subsplit between *a* and *d* must divide $U(\bar{s})$ under restriction. Finally, since the tips in $U(\bar{s})$ can only be partitioned once (in $\bar{s}$), every subsplit between *a* and *d* must be trivial under restriction.

We use the notation $q(a \rightarrow _*d)$ to represent the probability of observing subsplits *a* and *d* in a random tree from *q* if *d* is a valid descendant of *a* and zero otherwise. The unconditional probability of a PCSP under a restricted distribution is then,

$$\begin{aligned} q \mathop {\hspace{-1.66656pt}\downharpoonright \hspace{-1.111pt}\mathchoice{\hspace{-1.94443pt}}{\hspace{-1.94443pt}}{}{}_{\bar{X}}} \mathchoice{\hspace{-0.55542pt}}{\hspace{-0.55542pt}}{\hspace{-0.27771pt}}{} (\bar{t} \rightarrow \bar{s}) = 1_{\{{\bar{t}} \rightarrow {\bar{s}}\}} \sum _{a:\, a \mathop {\hspace{-1.66656pt}\downharpoonright \hspace{-1.111pt}\mathchoice{\hspace{-1.94443pt}}{\hspace{-1.94443pt}}{}{}_{\bar{X}}} \mathchoice{\hspace{-0.55542pt}}{\hspace{-0.55542pt}}{\hspace{-0.27771pt}}{} = \bar{t}} \, \sum _{d:\, d \mathop {\hspace{-1.66656pt}\downharpoonright \hspace{-1.111pt}\mathchoice{\hspace{-1.94443pt}}{\hspace{-1.94443pt}}{}{}_{\bar{X}}} \mathchoice{\hspace{-0.55542pt}}{\hspace{-0.55542pt}}{\hspace{-0.27771pt}}{} = \bar{s}} q(a \rightarrow _*d). \end{aligned}$$

Then under restriction, the conditional probability of a PCSP given its parent is

7 $$\begin{aligned} q \mathop {\hspace{-1.66656pt}\downharpoonright \hspace{-1.111pt}\mathchoice{\hspace{-1.94443pt}}{\hspace{-1.94443pt}}{}{}_{\bar{X}}} \mathchoice{\hspace{-0.55542pt}}{\hspace{-0.55542pt}}{\hspace{-0.27771pt}}{} (\bar{s} \vert \bar{t}) = \frac{ q \mathop {\hspace{-1.66656pt}\downharpoonright \hspace{-1.111pt}\mathchoice{\hspace{-1.94443pt}}{\hspace{-1.94443pt}}{}{}_{\bar{X}}} \mathchoice{\hspace{-0.55542pt}}{\hspace{-0.55542pt}}{\hspace{-0.27771pt}}{} ({\bar{t}} \rightarrow {\bar{s}})}{ q \mathop {\hspace{-1.66656pt}\downharpoonright \hspace{-1.111pt}\mathchoice{\hspace{-1.94443pt}}{\hspace{-1.94443pt}}{}{}_{\bar{X}}} \mathchoice{\hspace{-0.55542pt}}{\hspace{-0.55542pt}}{\hspace{-0.27771pt}}{} (\bar{t})}. \end{aligned}$$

### Supertree Support

Our overall goal is to find a distribution *q* on topologies that is close to a set of reference distributions $\{p_i\}$ on taxon sets $X_i$. An important part of that goal is to understand the *supertree support*, namely the set of building blocks (subsplits or PCSPs) that have positive probability under *q*, and is isomorphic to the set of trees that have positive probability under *q*. We find the supports for all of our reference distributions $p_i$, which we will call our *reference supports*. We will refer to process of finding a mutual supertree support for the entire taxon set as *mutualizing* or *mutualization*. The details of how this is done will depend on whether we are using a CCD or SCD parameterization.

We aim to construct a suitable supertree support for the sake of computational tractability, so we wish to have as few elements in our supertree support as reasonably possible. However, any tree that restricts to a tree in each reference support is as suitable for inclusion in the supertree support as any other, so we must include them all. We codify these objectives as a pair of Requirements, stated here generally and later more specifically in CCD and SCD contexts.

1. To allow an element into the supertree support, it must restrict to elements in each reference support,
2. Any tree that, under restriction, is a subset of every reference support, must be included in the supertree support.

For more than two reference supports, we propose an incremental approach for building the mutual support: we start with taxon set $X_1$ and its reference support, extend to $X_1 \cup X_2$ by mutualizing with the reference support for $X_2$, then continue to $(X_1 \cup X_2) \cup X_3$, etc. Thus we will only present an algorithm for the case of $X = X_1 \cup X_2$. The algorithms extend to finding supertree supports for multiple sets simultaneously, but the computations grow exponentially in the number of simultaneous supports (see Discussion).

#### CCD subsplit supports

Assume that we have reference subsplit supports ${\mathscr {C}}_{X_i}$ for each taxon subset $X_i \subset X$ and wish to find a good candidate subsplit support $M(\{{\mathscr {C}}_{X_i}\})$ for the supertree distribution $q(\tau )$. We now specialize the Requirements to the CCD case:

1. If $s \in M(\{{\mathscr {C}}_{X_i}\})$, then for each *i*, $s \mathop {\hspace{-1.66656pt}\downharpoonright \hspace{-1.111pt}\mathchoice{\hspace{-1.94443pt}}{\hspace{-1.94443pt}}{}{}_{X_i}} \mathchoice{\hspace{-0.55542pt}}{\hspace{-0.55542pt}}{\hspace{-0.27771pt}}{} \in {\mathscr {C}}_{X_i}$,
2. For every tree $\tau$ on *X* such that, for each *i*, $\tau \mathop {\hspace{-1.66656pt}\downharpoonright \hspace{-1.111pt}\mathchoice{\hspace{-1.94443pt}}{\hspace{-1.94443pt}}{}{}_{X_i}} \mathchoice{\hspace{-0.55542pt}}{\hspace{-0.55542pt}}{\hspace{-0.27771pt}}{} \subseteq {\mathscr {C}}_{X_i}$ then $\tau \subseteq M(\{{\mathscr {C}}_{X_i}\})$.

Requirement  1 says that any subsplit in the mutualized support must exist (after restriction) in each of the input subsplit supports. Requirement 2 says that any topology that appears (after restriction) in each of the restricted supports must be present in the mutualized support. These are in fact fairly strong constraints. For example, if reference supports do not agree on overlapping sets of taxa, then the supertree support can be too small or even empty. However, if the reference supports are restrictions of the true supertree support, or are supersets of the true support restrictions, then the mutualized support will cover the true support. Below we present Algorithm 1 which fulfills these requirements.

Next we explain the Requirements in more detail for the case of two reference supports. For any clade $W \subseteq X$, define ${\mathscr {C}}_{X}(W)$ as all subsplits in ${\mathscr {C}}_{X}$ that divide *W*, including the trivial subsplit $\{W, \emptyset \}$. In order for a subsplit *s* to meet Requirement  1, *s* must be a member of both ${\mathscr {C}}_{X_1}(W)$ and ${\mathscr {C}}_{X_2}(W)$ after restriction.

To find a collection of subsplits that satisfies Requirement 2, we take an iterative approach over possible clades from the root to the tips (Algorithm 1). Starting with the stack containing the clade *X* (the union of all tip sets), we iteratively pop the next clade *W*, and we consider every pairing of subsplits $s_1,s_2 = \{Y_1, Z_1\}, \{Y_2, Z_2\}$ from ${\mathscr {C}}_{X_1}(W) \times {\mathscr {C}}_{X_2}(W)$. For each pair, we generate a set of two potential subsplits, defining the $\boxtimes$ operator:

$$\begin{aligned} s_1 \boxtimes s_2 :=\left\{ \{Y_1 \cup Y_2, Z_1 \cup Z_2\}, \{Y_1 \cup Z_2, Z_1 \cup Y_2\} \right\} . \end{aligned}$$

Note that potential subsplits will frequently have taxa in both child clades, but we will exclude these invalid subsplits. We add each nontrivial valid subsplit $s \in s_1 \boxtimes s_2$ to the output, and we push each child clade of *s* to the stack of clades to consider if the clade is size two or larger and it has not been visited before.

**Algorithm 1:**
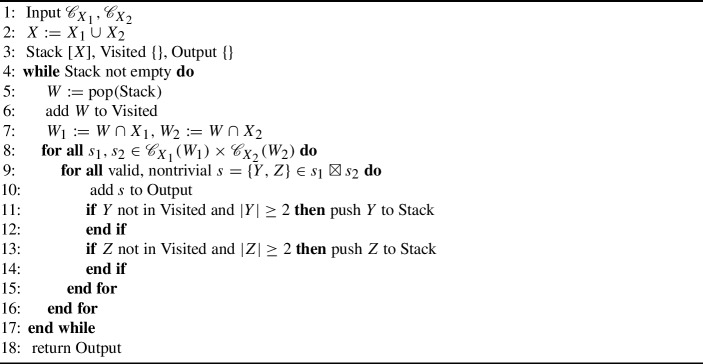
Mutual Subsplit Support algorithm $M({\mathscr {C}}_{X_1}, {\mathscr {C}}_{X_2})$

In the Appendix we prove that Algorithm 1 meets both of our requirements for the supertree subsplit support. We also show that Algorithm 1 runs in $O(n_{S_1} n_{S_2})$ time, where $n_{S_i}$ is the number of subsplits in reference support ${\mathscr {C}}_{X_i}$.

#### SCD PCSP Supports

For SCD-parameterized SBNs, we need to consider the supertree PCSP support. Suppose that we have reference PCSP supports ${\mathscr {P}}_{X_i}$ for each taxon subset $X_i \subset X$. We have requirements for PCSP support mutualization that parallel our requirements for constructing a subsplit support. Our Requirements take the following form in the SCD case:

1. If $({t}\rightarrow {s}) \in M(\{{\mathscr {P}}_{X_i}\})$, then for each *i*, there exists a path $(a_i \rightarrow _*t \rightarrow s) \subset M(\{{\mathscr {P}}_{X_i}\})$ and a subsplit $u_i$ in ${\mathscr {P}}_{X_i}$ such that $a_i \mathop {\hspace{-1.66656pt}\downharpoonright \hspace{-1.111pt}\mathchoice{\hspace{-1.94443pt}}{\hspace{-1.94443pt}}{}{}_{X_i}} \mathchoice{\hspace{-0.55542pt}}{\hspace{-0.55542pt}}{\hspace{-0.27771pt}}{} = u_i$ and $U( s \mathop {\hspace{-1.66656pt}\downharpoonright \hspace{-1.111pt}\mathchoice{\hspace{-1.94443pt}}{\hspace{-1.94443pt}}{}{}_{X_i}} \mathchoice{\hspace{-0.55542pt}}{\hspace{-0.55542pt}}{\hspace{-0.27771pt}}{} ) \in u_i$ (see Fig. 3 for an illustration).
2. For every tree $\tau$ on *X* such that, for each *i*, $\tau \mathop {\hspace{-1.66656pt}\downharpoonright \hspace{-1.111pt}\mathchoice{\hspace{-1.94443pt}}{\hspace{-1.94443pt}}{}{}_{X_i}} \mathchoice{\hspace{-0.55542pt}}{\hspace{-0.55542pt}}{\hspace{-0.27771pt}}{} \subseteq {\mathscr {P}}_{X_i}$ then $\tau \subseteq M(\{{\mathscr {P}}_{X_i}\})$.

**Fig. 4:**
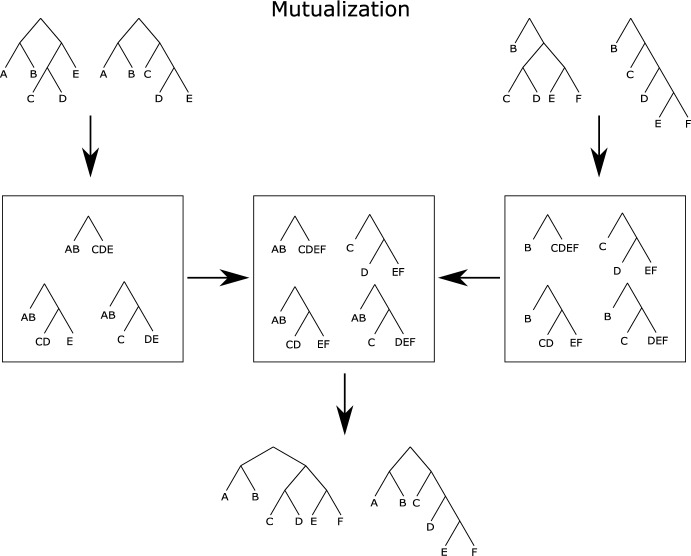
Illustration of finding the mutual PCSP support. On the top left and right we see the trees in the two reference distributions, on taxon sets $\{A, B, C, D, E\}$ and $\{B, C, D, E, F\}$, respectively. Below them (boxed), we see the PCSP supports resulting from those trees. In the center (boxed), we see the PCSP support on $\{A, B, C, D, E, F\}$ resulting from the mutualization algorithm. Below that, we see the trees that result from the mutual PCSP support. Note that all of the the trees at the bottom restrict to trees in the references

Our PCSP mutualization algorithm, laid out in Algorithm 2 and illustrated in Fig. 4, largely follows the structure of Algorithm 1, with a few subtle differences. We use the notation ${\mathscr {P}}_{X}(({t},\underline{W}))$ to represent the set of all valid child subsplits of $({t},\underline{W})$ in ${\mathscr {P}}_{X}$, including the trivial subsplit $\{W, \emptyset \}$. Because parent subsplits can become trivial under restriction, we need to perform additional bookkeeping in Algorithm 2. The items in our recursion stack (line 5) contain three pieces of information: the parent subsplit and clade under consideration in the full taxon set, the most recent parent subsplit in ${\mathscr {P}}_{X_1}$, and the most recent parent subsplit in ${\mathscr {P}}_{X_2}$. The additional **if** statements (lines 12 and 14) in the main loop capture both most recent parent subsplits in their respective supports. The need for and operation of these additional constructions is best illustrated with an example, which we provide in the Appendix. This example uses only 4 taxa and is written in great detail.

**Algorithm 2:**
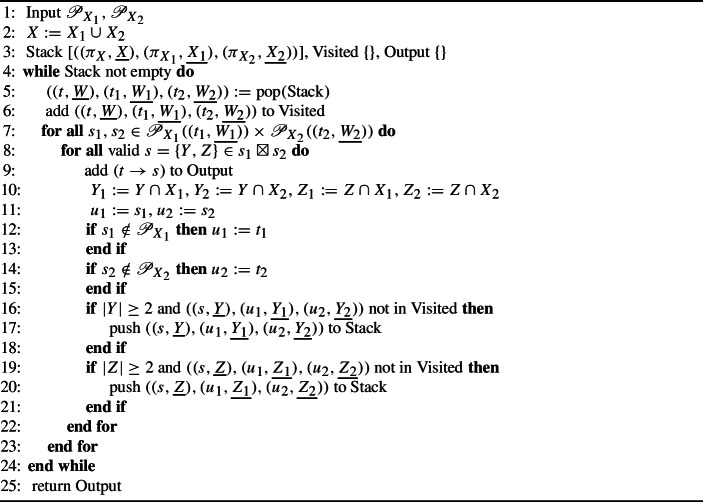
Mutual PCSP Support algorithm $M({\mathscr {P}}_{X_1}, {\mathscr {P}}_{X_2})$

In the Appendix we prove that Algorithm 2 meets both of our requirements for the supertree PCSP support. We also show that Algorithm 2 runs in $O(n_{P_1}, n_{P_2})$ time, where $n_{P_i}$ is the number of PCSPs in reference support ${\mathscr {P}}_{X_i}$.

### Gradients

**Fig. 5:**
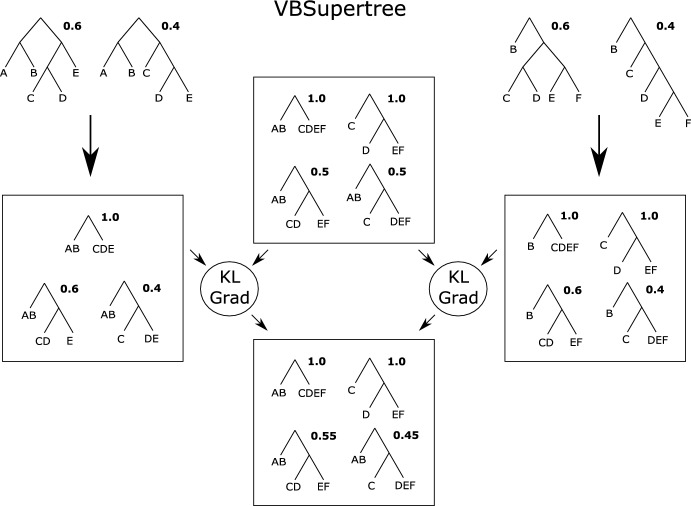
Illustration of the vbsupertree optimization loop. On the top left and top right we see the two reference distributions. Below them, we see the SBNs resulting from those trees (boxed). In the center (boxed, above) we see a candidate supertree SBN on trees in the mutual support. We calculate the KL-divergences and gradients versus the reference SBNs (under restriction) and change the candidate SBN according to the gradients, resulting in the next candidate SBN (center, below). The result can then be looped until a desired KL-divergence or a maximum number of iterations is reached

If we can calculate the gradient of $L(\{p_i\} \parallel q) = \sum _i D_{\text {KL}}(p_i \parallel q \mathop {\hspace{-1.66656pt}\downharpoonright \hspace{-1.111pt}\mathchoice{\hspace{-1.94443pt}}{\hspace{-1.94443pt}}{}{}_{X_i}} \mathchoice{\hspace{-0.55542pt}}{\hspace{-0.55542pt}}{\hspace{-0.27771pt}}{} )$ with respect to the parameters of our SBN, then we can use gradient descent to minimize our objective and optimize our supertree distribution. In this section we describe how to perform such gradient calculation in the CCD and the SCD parameterizations. We refer to our gradient-based approach as vbsupertree, and illustrate its optimization loop in Fig. 5.

#### CCD Parameterizations

Under CCDs, we parameterize the distribution of subsplits *s* conditional on their clade *U*(*s*) using a *softmax* transformation of a parameter vector ${\textbf{v}} = \{v_{s}\}$, i.e.

$$\begin{aligned} q(s \mid U(s)) = \frac{\exp ({v_{s}})}{\sum _{s':U(s')=U(s)}\exp ({v_{s'}})}. \end{aligned}$$

We choose this softmax parameterization in order to have clean derivatives with respect to our parameters and facilitate taking the gradient of our objective function. Softmax is also very commonly used in other statistical applications, so additional features like regularization penalties are easy to implement. We use the shorthand $\partial _{s}f({\textbf{v}}) = \frac{\partial }{\partial v_{s}} f({\textbf{v}})$ for the derivative of a function with respect to one of our CCD parameters.

Following the deriviations shown in the Appendix, we see that

$$\begin{aligned} \partial _{s'} D_{\text {KL}}(p \parallel q \mathop {\hspace{-1.66656pt}\downharpoonright \hspace{-1.111pt}\mathchoice{\hspace{-1.94443pt}}{\hspace{-1.94443pt}}{}{}_{\bar{X}}} \mathchoice{\hspace{-0.55542pt}}{\hspace{-0.55542pt}}{\hspace{-0.27771pt}}{} ) = \sum _s \left[ \frac{ p(U( s \mathop {\hspace{-1.66656pt}\downharpoonright \hspace{-1.111pt}\mathchoice{\hspace{-1.94443pt}}{\hspace{-1.94443pt}}{}{}_{\bar{X}}} \mathchoice{\hspace{-0.55542pt}}{\hspace{-0.55542pt}}{\hspace{-0.27771pt}}{} )) }{ q \mathop {\hspace{-1.66656pt}\downharpoonright \hspace{-1.111pt}\mathchoice{\hspace{-1.94443pt}}{\hspace{-1.94443pt}}{}{}_{\bar{X}}} \mathchoice{\hspace{-0.55542pt}}{\hspace{-0.55542pt}}{\hspace{-0.27771pt}}{} (U( s \mathop {\hspace{-1.66656pt}\downharpoonright \hspace{-1.111pt}\mathchoice{\hspace{-1.94443pt}}{\hspace{-1.94443pt}}{}{}_{\bar{X}}} \mathchoice{\hspace{-0.55542pt}}{\hspace{-0.55542pt}}{\hspace{-0.27771pt}}{} )) } - \frac{ p( s \mathop {\hspace{-1.66656pt}\downharpoonright \hspace{-1.111pt}\mathchoice{\hspace{-1.94443pt}}{\hspace{-1.94443pt}}{}{}_{\bar{X}}} \mathchoice{\hspace{-0.55542pt}}{\hspace{-0.55542pt}}{\hspace{-0.27771pt}}{} ) }{ q \mathop {\hspace{-1.66656pt}\downharpoonright \hspace{-1.111pt}\mathchoice{\hspace{-1.94443pt}}{\hspace{-1.94443pt}}{}{}_{\bar{X}}} \mathchoice{\hspace{-0.55542pt}}{\hspace{-0.55542pt}}{\hspace{-0.27771pt}}{} ( s \mathop {\hspace{-1.66656pt}\downharpoonright \hspace{-1.111pt}\mathchoice{\hspace{-1.94443pt}}{\hspace{-1.94443pt}}{}{}_{\bar{X}}} \mathchoice{\hspace{-0.55542pt}}{\hspace{-0.55542pt}}{\hspace{-0.27771pt}}{} ) } \right] \partial _{s'} q(s), \end{aligned}$$

where

$$\begin{aligned} \partial _{s'} q(s) = q(U(s')) q(s' \vert U(s')) \left[ q(s' \rightarrow _*s \mid s') - q(U(s') \rightarrow _*s \mid U(s'))\right] . \end{aligned}$$

This form clearly shows the algorithmic complexity of the gradient computation as $O({n_{s}}^2)$ where ${n_{s}}$ is the number of subsplits in the support, since both the summation and the derivative traverse every subsplit.

#### SCD Parameterizations

Under SCDs, we parameterize the distribution of child subsplits *s* conditional on their parent subsplit and clade $({t},\underline{U(s)})$ with parameter vector ${\textbf{v}} = \{v_{s \vert t}\}$, i.e.

$$\begin{aligned} q(s \vert ({t},\underline{U(s)})) = \frac{\exp ({v_{s \vert t}})}{\sum _{s':({t},\underline{U(s)})\rightarrow s'}\exp ({v_{s' \vert t}})}. \end{aligned}$$

We use the shorthand $\partial _{s \vert t}f({\textbf{v}}) = \frac{\partial }{\partial v_{s \vert t}} f({\textbf{v}})$ for the derivative of a function with respect to one of our SCD parameters.

Following the derivations shown in the Appendix, we see that

$$\begin{aligned} \partial _{s' \vert t'} D_{\text {KL}}(p \parallel q \mathop {\hspace{-1.66656pt}\downharpoonright \hspace{-1.111pt}\mathchoice{\hspace{-1.94443pt}}{\hspace{-1.94443pt}}{}{}_{\bar{X}}} \mathchoice{\hspace{-0.55542pt}}{\hspace{-0.55542pt}}{\hspace{-0.27771pt}}{} )&= \sum _{\bar{t}} k_{\bar{t}} \frac{p(\bar{t})}{ q \mathop {\hspace{-1.66656pt}\downharpoonright \hspace{-1.111pt}\mathchoice{\hspace{-1.94443pt}}{\hspace{-1.94443pt}}{}{}_{\bar{X}}} \mathchoice{\hspace{-0.55542pt}}{\hspace{-0.55542pt}}{\hspace{-0.27771pt}}{} (\bar{t})} \sum _{ a \mathop {\hspace{-1.66656pt}\downharpoonright \hspace{-1.111pt}\mathchoice{\hspace{-1.94443pt}}{\hspace{-1.94443pt}}{}{}_{\bar{X}}} \mathchoice{\hspace{-0.55542pt}}{\hspace{-0.55542pt}}{\hspace{-0.27771pt}}{} = \bar{t}} \partial _{s' \vert t'} q(a) \\&\quad -\sum _{(\bar{t} \rightarrow \bar{s})} \frac{p(\bar{t})}{ q \mathop {\hspace{-1.66656pt}\downharpoonright \hspace{-1.111pt}\mathchoice{\hspace{-1.94443pt}}{\hspace{-1.94443pt}}{}{}_{\bar{X}}} \mathchoice{\hspace{-0.55542pt}}{\hspace{-0.55542pt}}{\hspace{-0.27771pt}}{} (\bar{t})} \frac{p(\bar{s} \vert \bar{t})}{ q \mathop {\hspace{-1.66656pt}\downharpoonright \hspace{-1.111pt}\mathchoice{\hspace{-1.94443pt}}{\hspace{-1.94443pt}}{}{}_{\bar{X}}} \mathchoice{\hspace{-0.55542pt}}{\hspace{-0.55542pt}}{\hspace{-0.27771pt}}{} (\bar{s} \vert \bar{t})} \sum _{ a \mathop {\hspace{-1.66656pt}\downharpoonright \hspace{-1.111pt}\mathchoice{\hspace{-1.94443pt}}{\hspace{-1.94443pt}}{}{}_{\bar{X}}} \mathchoice{\hspace{-0.55542pt}}{\hspace{-0.55542pt}}{\hspace{-0.27771pt}}{} = \bar{t}} \sum _{ d \mathop {\hspace{-1.66656pt}\downharpoonright \hspace{-1.111pt}\mathchoice{\hspace{-1.94443pt}}{\hspace{-1.94443pt}}{}{}_{\bar{X}}} \mathchoice{\hspace{-0.55542pt}}{\hspace{-0.55542pt}}{\hspace{-0.27771pt}}{} = \bar{s}} \partial _{s' \vert t'} q(a \rightarrow _*d), \end{aligned}$$

where $k_{\bar{t}}$ is the number of child clades of $\bar{t}$ of size 2 or larger,

$$\begin{aligned} \partial _{s' \vert t'} q(a) =&\, q(t') {\mathcal {D}}_{q}(s' \vert t'; a), \\ \partial _{s' \vert t'} q(a \rightarrow _*d) =&\, q(a) q(a \rightarrow _*t' \vert a) {\mathcal {D}}_{q}(s' \vert t'; d) + q(t') {\mathcal {D}}_{q}(s' \vert t'; a) q(a \rightarrow _*d \vert a), \text { and}\\ {\mathcal {D}}_{q}(s' \vert t'; a) :=&\, q(s' \vert ({t'},\underline{U(s')})) \left[ q(s' \rightarrow _*a \mid s') - q(({t'},\underline{U(s')}) \rightarrow _*a \mid ({t'},\underline{U(s')})) \right] . \end{aligned}$$

After a linear pass through the support accumulating path probabilities, the algorithmic efficiency of this calculation is $O(n_{p}\cdot n_{p} \mathop {\hspace{-1.66656pt}\downharpoonright \hspace{-1.111pt}\mathchoice{\hspace{-1.94443pt}}{\hspace{-1.94443pt}}{}{}_{\bar{X}}} \mathchoice{\hspace{-0.55542pt}}{\hspace{-0.55542pt}}{\hspace{-0.27771pt}}{} )$, where $n_{p}$ is the number of PCSPs in the support, and $n_{p} \mathop {\hspace{-1.66656pt}\downharpoonright \hspace{-1.111pt}\mathchoice{\hspace{-1.94443pt}}{\hspace{-1.94443pt}}{}{}_{\bar{X}}} \mathchoice{\hspace{-0.55542pt}}{\hspace{-0.55542pt}}{\hspace{-0.27771pt}}{}$ is the number of paths in the support that restrict to a PCSP on tip set $\bar{X}$.

## Results

### Simulated Data

We begin exploring the effectiveness of vbsupertree through a simulation study. We sample a phylogenetic tree with 40 tips from the classical isochronous, constant effective population size coalescent, and simulate a sequence data alignment using the Jukes-Cantor 1969 model (Jukes and Cantor 1969). We remove one sequence from the full alignment to make our first reference alignment, and remove a different sequence to create our second reference alignment. We approximate the posterior tree distributions for our full alignment and our two reference alignments by running Markov chain Monte Carlo (Hastings 1970) using the phylogenetic software BEAST (Drummond and Rambaut 2007; Suchard et al. 2018) on our three sequence datasets. We run BEAST for $10^7$ steps, remove 50% burn-in from the beginning, and subsample every 1000th tree to reduce autocorrelation, resulting in a 5000 tree posterior samples. Since we are not guaranteed to see every credible tree topology in every run due to the size of tree space, to maintain support compatibility for KL-divergence calculations, we trim out all tree topologies that only appear in a given BEAST output once, in order to increase the proportion of PCSPs in common (under the appropriate restriction) between the three posterior samples. We train rooted, SCD-parameterized SBNs to use as our ground truth distribution and two reference distributions. Finally, we trim any PCSPs in our ground truth and our references that are not covered by the appropriate restriction of our mutualized support (no restriction in the case of the ground truth).

**Fig. 6:**
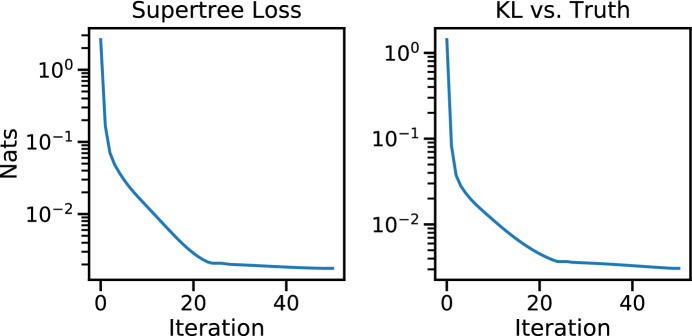
The progression of the loss function (left panel) and the progression of the KL-divergence versus the truth (right panel) over 50 iterations of vbsupertree applied to simulated data

Applying vbsupertree to the two generated reference distribution leads to quick convergence of the loss function to a small value, as seen in Fig. 6, left panel. Additionally, knowing a sample of the true posterior, we chart the progression of the KL-divergence of our supertree SBN versus the truth (SBN trained on the full posterior), resulting in the right panel.

### Real World Data

For an analysis on real world data, we select 30 well-differentiated hepatitis C virus (HCV) sequences from the alignment previously analyzed by Pybus et al. (2003) and others. We remove one sequence from the full alignment to make our first reference alignment, and remove a different sequence to create our second reference alignment. From this stage forward, our approach is identical to our simulation study. We approximate the true posterior and our two references by running BEAST on our three sequence datasets. We run BEAST for $10^9$ steps, remove 50% burn-in from the beginning, and subsample every 5000th tree to reduce autocorrelation, resulting in a 100000 tree posterior samples. In order to make the supports compatible for KL-divergence calculations, we trim out all tree topologies that only appear in a given BEAST output once, We train rooted, SCD-parameterized SBNs to use as our ground truth distribution and two reference distributions. Finally, we trim any PCSPs in our ground truth and our references that are not covered by the appropriate restriction of our mutualized support.

**Fig. 7:**
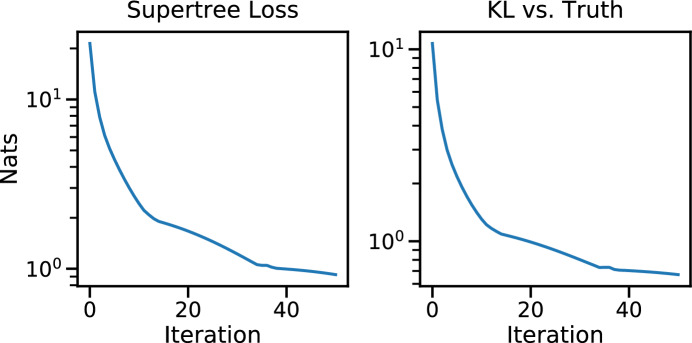
The progression of the loss function (left panel) and the progression of the KL-divergence versus the truth (right panel) over 50 iterations of vbsupertree applied to the HCV dataset

Applying vbsupertree to the two generated reference distribution leads to quick convergence of the loss function to a small value, as seen in Fig. 7, left panel. Additionally, knowing a sample of the true posterior, we chart the progression of the KL-divergence of our supertree SBN versus the truth (SBN trained on the full posterior), resulting in the right panel.

## Discussion

In this paper, we lay out an SBN-based framework for generating supertree supports and training variational supertree distributions. We apply our method to simulated sequence data and find that it trains an SBN that very closely approximates our target posterior distribution. We also apply our method to a subset of a well-known HCV dataset, and successfully train it to approximate our ground truth distribution.

Although the work of Ronquist et al. (2004) described in the introduction is the closest work to that presented here, two other lines of research deserve mention in this context. First, De Oliveira et al. (2016) derive a Bayesian extension of previous work on maximum likelihood supertrees (Steel and Rodrigo 2008). In this strategy, one posits a likelihood model based on measures of disagreement between trees, such as an exponential likelihood in terms of some distance between tree topologies. This method is interesting in that it can incorporate a number of distances representing various aspects of tree disagreement (De Oliveira et al. 2016), however, this is a different than the direct goal of reconstructing a posterior distribution on taxon set *X* given its projections onto subsets as we describe below. Our objective directly phrases a goal appropriate for divide-and-conquer Bayesian phylogenetics. Also, the work of Bryant (2001) shares some goals and concepts with our mutualization algorithm. However, in that setting one has one tree per taxon subset (rather than many as is the case here) and one wishes to find the optimal tree among the potentially many trees that restrict to the stated tree for each taxon subset.

Another related line of research concerns sequential Monte Carlo inference by subtree merging (Bouchard-Côté et al. 2012; Wang et al. 2015). The state of such a sampler is described by a population of “particles,” each of which consists of a collection of rooted trees on disjoint taxon subsets such that the union of the tree tips is the entire taxon set. In each step of the algorithm, particles are chosen from the previous generation, and for each particle a pair of subtrees are merged. These probabilistic choices and mergings are designed carefully such that after completion of all of the steps one obtains a sample from the phylogenetic posterior distribution. This method is in a sense a type of divide-and-conquer algorithm in that it finds solutions to phylogenetic problems on subsets of taxa before finding the entire posterior. However, it differs significantly from our current goal in that we assume that the taxon subsets and the posterior distributions on subtrees are delivered as part of the problem statement, whereas phylogenetic SMC ingests raw molecular sequence data.

One common obstacle for supertree methods is the fact that the compatibility of *k* tree topologies on *k* tip sets cannot be checked in polynomial time in *k* (Steel 1992). For the methods we present, holding ourselves to two tip sets at a time, this is not an issue. Our subsplit- and PCSP-based approaches pool our topologies into two sets which effectively sets $k=2$. It is for this reason we propose using a one-at-a-time approach for using our supertree support mutualization methods on $k>2$ tip sets. We anticipate exploring the properties of one-at-a-time versus all-at-once mutualization in future work.

The $k=2$ framework may also have its own “Curse of Dimensionality” if the reference tip sets have little overlap. In this case, the information content of the reference distributions might be very small compared to the dimensionality of the supertree distribution, due to the rapid expansion of tree space on the number of tips. In these cases, the choice of the initial supertree distribution (before gradient descent) may influence the final (converged) supertree distribution. In our main experiments, we have taken the approach of using high-entropy starting distributions, but in Appendix A.6 we incorporate a regularization penalty term, to encourage conservative conditional distributions where the information from the reference distributions is insufficient.

One caveat for our supertree support mutualization methods arises when the reference supports do not completely cover the true restricted supports. When the references cover the truth, our results guarantee that the mutualized support contains every topology that we require without containing any extraneous elements. However, if the reference supports are missing elements from the true supports, then topologies will go missing from the mutualized supertree support and it is not guaranteed to cover the true support. Unfortunately, most tree-based Bayesian analyses will have enormous posterior topology supports, and Monte Carlo based methods will collect only a sample from the larger posterior. Thus in future work, we intend to broaden the inclusion criteria for our supertree support methods while still attempting to keep the mutual support as small as possible.

In general we view this work as providing a proof of concept of a new approach for divide-and-conquer Bayesian phylogenetics. To make this a more complete method, it will also require methods to merge variational branch length distributions (Zhang and Matsen IV 2019). Further refinement of these merged distributions with the complete data set, in terms of both support and continuous parameters, will likely be required. We also note that a perfect estimate of the variational distribution on topologies may not be necessary, as one can correct these variational distributions using importance sampling (Zhang and Matsen IV 2019), or perhaps use them as part of an MCMC proposal. However, high-quality importance sampling may require larger topology supports, potentially up to the full tree space for a given tip set. In that case, our mutualization and supertree methods can be adapted to include a nonzero probability for topologies outside of the mutual support. Future work will include exploration of the quality of importance sampling approximations using SBNs, including exploration of issues of support size and compatibility.

## Figures and Tables

**Fig. 8:**
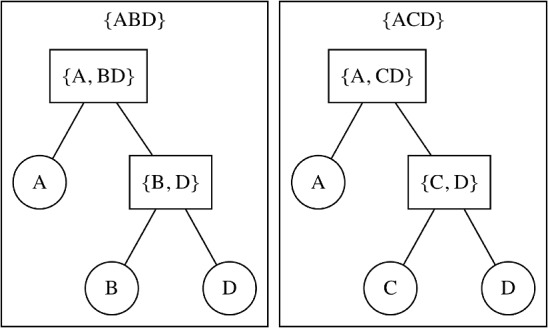
Example starting trees for finding the mutual PCSP support

**Fig. 9:**
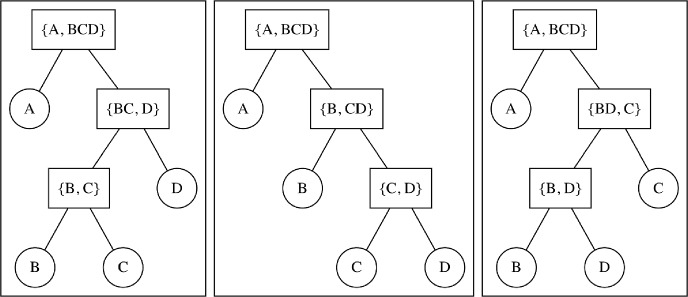
Trees resulting from the mutual PCSP algorithm example applied to the trees from Fig. 8

**Fig. 10:**
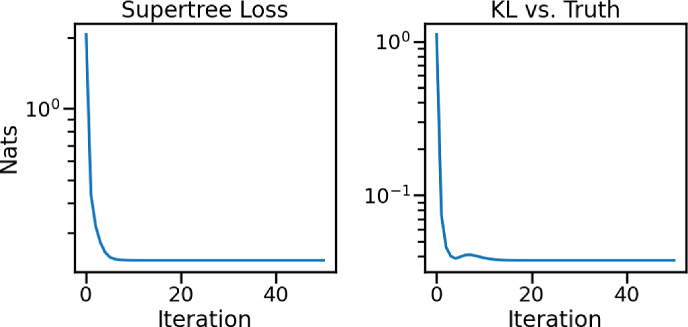
The progression of the loss function (left panel) and the progression of the KL-divergence versus the truth (right panel) over 50 iterations of vbsupertree applied to simulated data with penalty 0.05

**Fig. 11:**
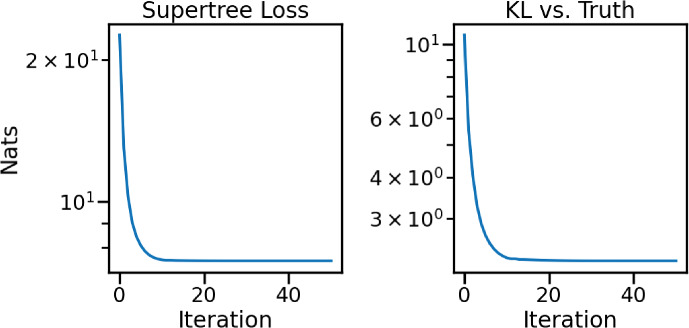
The progression of the loss function (left panel) and the progression of the KL-divergence versus the truth (right panel) over 50 iterations of vbsupertree applied to the HCV dataset with penalty 0.05

**Fig. 12:**
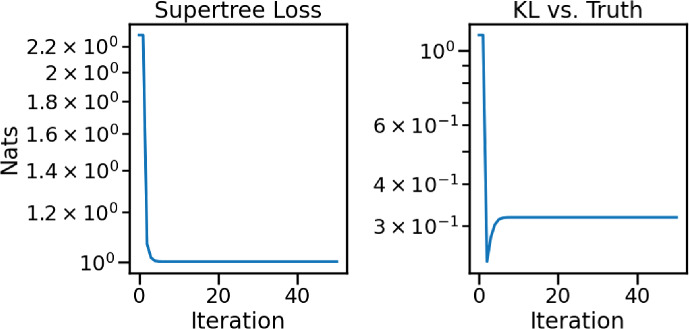
The progression of the loss function (left panel) and the progression of the KL-divergence versus the truth (right panel) over 50 iterations of vbsupertree applied to simulated data with penalty 0.50

**Fig. 13:**
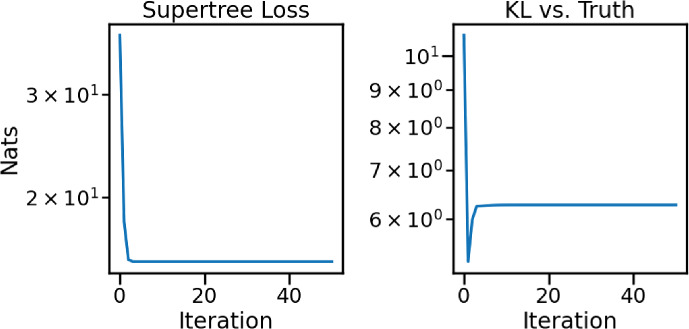
The progression of the loss function (left panel) and the progression of the KL-divergence versus the truth (right panel) over 50 iterations of vbsupertree applied to the HCV dataset with penalty 0.50

## Data Availability

Data and materials are supplied as an online supplement (zipped file) and can be found here https://doi.org/10.5281/zenodo.4793979.

## Appendix A Proofs and Examples

### CCD Subsplit Support Proofs

Our first theorem establishes that Algorithm 1 satisfies CCD Requirement  1.

#### Theorem 1

$$ M({\mathscr {C}}_{X_1}, {\mathscr {C}}_{X_2}) \mathop {\hspace{-1.66656pt}\downharpoonright \hspace{-1.111pt}\mathchoice{\hspace{-1.94443pt}}{\hspace{-1.94443pt}}{}{}_{X_1}} \mathchoice{\hspace{-0.55542pt}}{\hspace{-0.55542pt}}{\hspace{-0.27771pt}}{} \subseteq {\mathscr {C}}_{X_1}$$ and $$ M({\mathscr {C}}_{X_1}, {\mathscr {C}}_{X_2}) \mathop {\hspace{-1.66656pt}\downharpoonright \hspace{-1.111pt}\mathchoice{\hspace{-1.94443pt}}{\hspace{-1.94443pt}}{}{}_{X_2}} \mathchoice{\hspace{-0.55542pt}}{\hspace{-0.55542pt}}{\hspace{-0.27771pt}}{} \subseteq {\mathscr {C}}_{X_2}$$.

#### Proof

By construction, every subsplit $s \in M({\mathscr {C}}_{X_1}, {\mathscr {C}}_{X_2})$ $X_1$-restricts to a subsplit $s \mathop {\hspace{-1.66656pt}\downharpoonright \hspace{-1.111pt}\mathchoice{\hspace{-1.94443pt}}{\hspace{-1.94443pt}}{}{}_{X_1}} \mathchoice{\hspace{-0.55542pt}}{\hspace{-0.55542pt}}{\hspace{-0.27771pt}}{} \in {\mathscr {C}}_{X_1}$ and $X_2$-restricts to a subsplit $s \mathop {\hspace{-1.66656pt}\downharpoonright \hspace{-1.111pt}\mathchoice{\hspace{-1.94443pt}}{\hspace{-1.94443pt}}{}{}_{X_2}} \mathchoice{\hspace{-0.55542pt}}{\hspace{-0.55542pt}}{\hspace{-0.27771pt}}{} \in {\mathscr {C}}_{X_2}$. $\square$

#### Lemma 2

Suppose there exists a tree topology $\tau$ with taxon set *X* such that $\tau \mathop {\hspace{-1.66656pt}\downharpoonright \hspace{-1.111pt}\mathchoice{\hspace{-1.94443pt}}{\hspace{-1.94443pt}}{}{}_{X_1}} \mathchoice{\hspace{-0.55542pt}}{\hspace{-0.55542pt}}{\hspace{-0.27771pt}}{} \subseteq {\mathscr {C}}_{X_1}$ and $\tau \mathop {\hspace{-1.66656pt}\downharpoonright \hspace{-1.111pt}\mathchoice{\hspace{-1.94443pt}}{\hspace{-1.94443pt}}{}{}_{X_2}} \mathchoice{\hspace{-0.55542pt}}{\hspace{-0.55542pt}}{\hspace{-0.27771pt}}{} \subseteq {\mathscr {C}}_{X_2}$. If $s \in \tau$ and the algorithm reaches state $W = U(s)$, then $s \in M({\mathscr {C}}_{X_1}, {\mathscr {C}}_{X_2})$.

#### Proof

Suppose subsplit $s = \{Y, Z\}$, then we know $s \mathop {\hspace{-1.66656pt}\downharpoonright \hspace{-1.111pt}\mathchoice{\hspace{-1.94443pt}}{\hspace{-1.94443pt}}{}{}_{X_1}} \mathchoice{\hspace{-0.55542pt}}{\hspace{-0.55542pt}}{\hspace{-0.27771pt}}{} = \{Y \cap X_1, Z \cap X_1\}$ and $s \mathop {\hspace{-1.66656pt}\downharpoonright \hspace{-1.111pt}\mathchoice{\hspace{-1.94443pt}}{\hspace{-1.94443pt}}{}{}_{X_2}} \mathchoice{\hspace{-0.55542pt}}{\hspace{-0.55542pt}}{\hspace{-0.27771pt}}{} = \{Y \cap X_2, Z \cap X_2\}$. By assumption $W = U(s)$, $s \mathop {\hspace{-1.66656pt}\downharpoonright \hspace{-1.111pt}\mathchoice{\hspace{-1.94443pt}}{\hspace{-1.94443pt}}{}{}_{X_1}} \mathchoice{\hspace{-0.55542pt}}{\hspace{-0.55542pt}}{\hspace{-0.27771pt}}{} \in {\mathscr {C}}_{X_1}$, and $s \mathop {\hspace{-1.66656pt}\downharpoonright \hspace{-1.111pt}\mathchoice{\hspace{-1.94443pt}}{\hspace{-1.94443pt}}{}{}_{X_2}} \mathchoice{\hspace{-0.55542pt}}{\hspace{-0.55542pt}}{\hspace{-0.27771pt}}{} \in {\mathscr {C}}_{X_2}$, so the algorithm considers subsplits in ${\mathscr {C}}_{X_1}$ that divide $W_1 :=W \cap X_1$, and subsplits in ${\mathscr {C}}_{X_2}$ that divide $W_2 :=W \cap X_2$. We know

$$\begin{aligned} W_1&= W \cap X_1 = U(s) \cap X_1 = (Y \cup Z) \cap X_1 \\&= (Y \cap X_1) \cup (Z \cap X_1) = U( s \mathop {\hspace{-1.66656pt}\downharpoonright \hspace{-1.111pt}\mathchoice{\hspace{-1.94443pt}}{\hspace{-1.94443pt}}{}{}_{X_1}} \mathchoice{\hspace{-0.55542pt}}{\hspace{-0.55542pt}}{\hspace{-0.27771pt}}{} ). \end{aligned}$$

Similarly, $W_2 = U( s \mathop {\hspace{-1.66656pt}\downharpoonright \hspace{-1.111pt}\mathchoice{\hspace{-1.94443pt}}{\hspace{-1.94443pt}}{}{}_{X_2}} \mathchoice{\hspace{-0.55542pt}}{\hspace{-0.55542pt}}{\hspace{-0.27771pt}}{} )$, so the algorithm considers $s \mathop {\hspace{-1.66656pt}\downharpoonright \hspace{-1.111pt}\mathchoice{\hspace{-1.94443pt}}{\hspace{-1.94443pt}}{}{}_{X_1}} \mathchoice{\hspace{-0.55542pt}}{\hspace{-0.55542pt}}{\hspace{-0.27771pt}}{}$ from ${\mathscr {C}}_{X_1}$ and $s \mathop {\hspace{-1.66656pt}\downharpoonright \hspace{-1.111pt}\mathchoice{\hspace{-1.94443pt}}{\hspace{-1.94443pt}}{}{}_{X_2}} \mathchoice{\hspace{-0.55542pt}}{\hspace{-0.55542pt}}{\hspace{-0.27771pt}}{}$ from ${\mathscr {C}}_{X_2}$ at this step. Then one of the subsplits on *X* that the algorithm generates is

$$\begin{aligned} \{[Y \cap X_1] \cup [Y \cap X_2], [Z \cap X_1] \cup [Z \cap X_2]\}&= \{Y \cap [X_1 \cup X_2], Z \cap [X_1 \cup X_2]\} \\&= \{Y \cap X, Z \cap X\} = s. \end{aligned}$$

We know *s* is valid and nontrivial, so $s \in M({\mathscr {C}}_{X_1}, {\mathscr {C}}_{X_2})$. $\square$

We can now establish that Algorithm 1 satisfies CCD Requirement 2.

#### Theorem 3

If there exists a tree topology $\tau$ with taxon set *X* such that $\tau \mathop {\hspace{-1.66656pt}\downharpoonright \hspace{-1.111pt}\mathchoice{\hspace{-1.94443pt}}{\hspace{-1.94443pt}}{}{}_{X_1}} \mathchoice{\hspace{-0.55542pt}}{\hspace{-0.55542pt}}{\hspace{-0.27771pt}}{} \subseteq {\mathscr {C}}_{X_1}$ and $\tau \mathop {\hspace{-1.66656pt}\downharpoonright \hspace{-1.111pt}\mathchoice{\hspace{-1.94443pt}}{\hspace{-1.94443pt}}{}{}_{X_2}} \mathchoice{\hspace{-0.55542pt}}{\hspace{-0.55542pt}}{\hspace{-0.27771pt}}{} \subseteq {\mathscr {C}}_{X_2}$, then $\tau \subseteq M({\mathscr {C}}_{X_1}, {\mathscr {C}}_{X_2})$.

#### Proof

We use a proof by induction recursively from root to tips over the subsplits in $\tau$. Our base case is the root split of $\tau$. The algorithm begins with $W = X$ in the stack, so by Lemma 2$\tau$’s root split will be in the output. Our inductive step for general $s \in \tau$ allows us to assume that *s*’s parent $\pi _{\tau }{(s)}$ has already been visited and is already in the output. Since $\pi _{\tau }{(s)} \rightarrow s$ and $s \in \tau$, we know $U(s) \in \pi _{\tau }{(s)}$ and $|U(s) |> 1$, so *U*(*s*) will already be in the stack and the algorithm will eventually reach state $W = U(s)$. Then by Lemma 2*s* will be in the output. Therefore, by induction, all $s \in \tau$ are in $M({\mathscr {C}}_{X_1}, {\mathscr {C}}_{X_2})$, so $\tau \subseteq M({\mathscr {C}}_{X_1}, {\mathscr {C}}_{X_2})$. $\square$

Finally, we show that Algorithm 1 runs in $O(n_{S_1} n_{S_2})$ time, where $n_{S_i}$ is the number of subsplits in reference support ${\mathscr {C}}_{X_i}$. We know Algorithm 1 only visits each clade $W \in X$ at most once, each *W* restricts to one pair of $W_1, W_2$, and each subsplit divides only one clade. In the worst case, where $X_1$ and $X_2$ are disjoint, any $W_1$ may be paired with any $W_2$, so every subsplit in ${\mathscr {C}}_{X_1}$ might have to be crossed with every subsplit in ${\mathscr {C}}_{X_2}$. Therefore the algorithm runs in $O(n_{S_1} n_{S_2})$ time.

### Mutual PCSP Support Example

We will now illustrate Algorithm 2 with a simple yet nontrivial example. To illustrate specific clades, subsplits, and PCSPs, we introduce some shorthand notations for this section. Tree tips will be represented by capital letters, such as $\texttt{A}$ and $\texttt{B}$. Clades will be represented by concatenated tips, such as $\texttt{ABD}$ and $\texttt{ACD}$. Subsplits will be represented by two clades in a set, such as $\{\mathtt {{AB},{CD}}\}$. Subsplits focusing on a specific child clade will be represented by two clades in a set, with the focus clade underlined and generally written second, such as $\{\mathtt {{CD},\underline{AB}}\}$. PCSPs will then look like $\{\mathtt {{CD},\underline{AB}}\} \rightarrow \{\mathtt {{A},{B}}\}$.

Suppose we are given the trees depicted in Fig. 8. The tree with tips $\texttt{ABD}$ results in the reference PCSP support

$$\begin{aligned} {\mathscr {P}}_{\texttt{ABD}} = \{\{\mathtt {{\emptyset },\underline{ABD}}\}&\rightarrow \{\mathtt {{A},{BD}}\},\\ \{\mathtt {{A},\underline{BD}}\}&\rightarrow \{\mathtt {{B},{D}}\}\}, \end{aligned}$$

and the tree with tips $\texttt{ACD}$ results in

$$\begin{aligned} {\mathscr {P}}_{\texttt{ACD}} = \{\{\mathtt {{\emptyset },\underline{ACD}}\}&\rightarrow \{\mathtt {{A},{CD}}\},\\ \{\mathtt {{A},\underline{CD}}\}&\rightarrow \{\mathtt {{C},{D}}\}\}, \end{aligned}$$

omitting singletons and the empty subsplit for brevity.

The union of the tip sets is $\texttt{ABCD}$. The mutual PCSP support algorithm begins with stack $[(\{\mathtt {{\emptyset },\underline{ABCD}}\}, \{\mathtt {{\emptyset },\underline{ABD}}\}, \{\mathtt {{\emptyset },\underline{ACD}}\})]$.

We consider the children (including the trivial child) of $\{\mathtt {{\emptyset },\underline{ABD}}\}$ which are $\{\{\mathtt {{A},{BD}}\}, \{\mathtt {{\emptyset },{ABD}}\}\}$ and the children of $\{\mathtt {{\emptyset },\underline{ACD}}\}$, namely $\{\{\mathtt {{A},{CD}}\}, \{\mathtt {{\emptyset },{ACD}}\}\}$. The only nontrivial subsplit this results in via $\boxtimes$ is $\{\mathtt {{A},{BCD}}\}$.

We add $\{\mathtt {{\emptyset },\underline{ABCD}}\} \rightarrow \{\mathtt {{A},{BCD}}\}$ to the output.

We see $\{\mathtt {{A},{BCD}}\}$ restricts to $\{\mathtt {{A},{BD}}\}$ and $\{\mathtt {{A},{CD}}\}$, both of which are subsplits in their respective reference supports. The only child clade of $\{\mathtt {{A},{BCD}}\}$ that is size 2 or larger is $\texttt{BCD}$, so we push $(\{\mathtt {{A},\underline{BCD}}\}, \{\mathtt {{A},\underline{BD}}\}, \{\mathtt {{A},\underline{CD}}\})$ to the stack.

We pop $$(\{\mathtt {{A},\underline{BCD}}\}, \{\mathtt {{A},\underline{BD}}\}, \{\mathtt {{A},\underline{CD}}\})$$.

We combine the subsplits $\{\{\mathtt {{B},{D}}\}, \{\mathtt {{\emptyset },{BD}}\}\}$ and $\{\{\mathtt {{C},{D}}\}, \{\mathtt {{\emptyset },{CD}}\}\}$ via $\boxtimes$ as before, resulting in a set of potential children: $\{\{\mathtt {{BC},{D}}\}, \{\mathtt {{B},{CD}}\}, \{\mathtt {{BD},{C}}\}\}$.

We add $\{\mathtt {{A},\underline{BCD}}\} \rightarrow \{\mathtt {{BC},{D}}\}$ to the output.

We see $\{\mathtt {{BC},{D}}\}$ restricts to $\{\mathtt {{B},{D}}\}$ and $\{\mathtt {{C},{D}}\}$, so we push $(\{\mathtt {{D},\underline{BC}}\}, \{\mathtt {{D},\underline{B}}\}, \{\mathtt {{D},\underline{C}}\})$ to the stack.

We add $\{\mathtt {{A},\underline{BCD}}\} \rightarrow \{\mathtt {{B},{CD}}\}$ to the output.

We see $\{\mathtt {{BC},{D}}\}$ restricts to $\{\mathtt {{B},{D}}\}$ and $\{\mathtt {{\emptyset },{CD}}\}$. However, $\{\mathtt {{\emptyset },{CD}}\}$ is not in ${\mathscr {P}}_{\texttt{ACD}}$, triggering the “else” clause, so we push $(\{\mathtt {{B},\underline{CD}}\}, \{\mathtt {{B},\underline{D}}\}, \{\mathtt {{A},\underline{CD}}\})$ to the stack.

We add $\{\mathtt {{A},\underline{BCD}}\} \rightarrow \{\mathtt {{BD},{C}}\}$ to the output.

We see $\{\mathtt {{BD},{C}}\}$ restricts to $\{\mathtt {{\emptyset },{BD}}\}$ (not in ${\mathscr {P}}_{ABD}$) and $\{\mathtt {{C},{D}}\}$, so we push $(\{\mathtt {{C},\underline{BD}}\}, \{\mathtt {{A},\underline{BD}}\}, \{\mathtt {{C},\underline{D}}\})$ to the stack.

We pop $$(\{\mathtt {{C},\underline{BD}}\}, \{\mathtt {{A},\underline{BD}}\}, \{\mathtt {{C},\underline{D}}\})$$.

We combine the subsplits $\{\{\mathtt {{B},{D}}\}, \{\mathtt {{\emptyset },{BD}}\}\}$ and $\{\{\mathtt {{\emptyset },{D}}\}\}$, resulting in $\{\mathtt {{B},{D}}\}$.

We add $\{\mathtt {{C},\underline{BD}}\} \rightarrow \{\mathtt {{B},{D}}\}$ to the output. No child clades are size 2 or larger, so we do not push anything to the stack.

We pop $$(\{\mathtt {{B},\underline{CD}}\}, \{\mathtt {{B},\underline{D}}\}, \{\mathtt {{A},\underline{CD}}\})$$.

We combine the subsplits $\{\{\mathtt {{\emptyset },{D}}\}\}$ and $\{\{\mathtt {{C},{D}}\}, \{\mathtt {{\emptyset },{CD}}\}\}$, resulting in $\{\mathtt {{C},{D}}\}$.

We add $\{\mathtt {{B},\underline{CD}}\} \rightarrow \{\mathtt {{C},{D}}\}$ to the output.

We pop $$(\{\mathtt {{D},\underline{BC}}\}, \{\mathtt {{D},\underline{B}}\}, \{\mathtt {{D},\underline{C}}\})$$.

We combine the subsplits $\{\{\mathtt {{\emptyset },{B}}\}\}$ and $\{\{\mathtt {{\emptyset },{C}}\}\}$, resulting in $\{\mathtt {{B},{C}}\}$.

We add $\{\mathtt {{D},\underline{BC}}\} \rightarrow \{\mathtt {{B},{C}}\}$ to the output.

The stack is empty, so the algorithm terminates here. The final output is the PCSP support

$$\begin{aligned} \{&\{\mathtt {{\emptyset },\underline{ABCD}}\} \rightarrow \{\mathtt {{A},{BCD}}\}, \\&\{\mathtt {{A},\underline{BCD}}\} \rightarrow \{\mathtt {{BC},{D}}\}, \\&\{\mathtt {{A},\underline{BCD}}\} \rightarrow \{\mathtt {{B},{CD}}\}, \\&\{\mathtt {{A},\underline{BCD}}\} \rightarrow \{\mathtt {{BD},{C}}\}, \\&\{\mathtt {{C},\underline{BD}}\} \rightarrow \{\mathtt {{B},{D}}\}, \\&\{\mathtt {{B},\underline{CD}}\} \rightarrow \{\mathtt {{C},{D}}\}, \\&\{\mathtt {{D},\underline{BC}}\} \rightarrow \{\mathtt {{B},{C}}\}\}. \end{aligned}$$

This PCSP support results in the trees depicted in Fig. 9. Note that each tree restricts to the appropriate reference trees in Fig. 8, as required.

### SCD PCSP Support Proofs

Here we prove that Algorithm 2 meets both of our requirements for the PCSP subsplit support. This first theorem establishes the first Requirement in the SCD case.

#### Theorem 4

For every $(t \rightarrow s) \in M({\mathscr {P}}_{X_1}, {\mathscr {P}}_{X_2})$ and each $i = 1,2$, there exists a path $(a_i \rightarrow _*t \rightarrow s) \subseteq M({\mathscr {P}}_{X_1}, {\mathscr {P}}_{X_2})$ and a subsplit $u_i$ in ${\mathscr {P}}_{X_i}$ such that $a_i \mathop {\hspace{-1.66656pt}\downharpoonright \hspace{-1.111pt}\mathchoice{\hspace{-1.94443pt}}{\hspace{-1.94443pt}}{}{}_{X_i}} \mathchoice{\hspace{-0.55542pt}}{\hspace{-0.55542pt}}{\hspace{-0.27771pt}}{} = u_i$ and $U( s \mathop {\hspace{-1.66656pt}\downharpoonright \hspace{-1.111pt}\mathchoice{\hspace{-1.94443pt}}{\hspace{-1.94443pt}}{}{}_{X_i}} \mathchoice{\hspace{-0.55542pt}}{\hspace{-0.55542pt}}{\hspace{-0.27771pt}}{} ) \in u_i$.

#### Proof

For any parent subsplit *t*, every candidate child subsplit *s* is constructed from the children (possibly trivial) of the most recent ancestors present in their respective reference support $\{ a_i \mathop {\hspace{-1.66656pt}\downharpoonright \hspace{-1.111pt}\mathchoice{\hspace{-1.94443pt}}{\hspace{-1.94443pt}}{}{}_{X_i}} \mathchoice{\hspace{-0.55542pt}}{\hspace{-0.55542pt}}{\hspace{-0.27771pt}}{} = u_i\}$. By induction, we know $a_i \rightarrow _*t$. Finally, by construction $t \rightarrow s$, $a_i \mathop {\hspace{-1.66656pt}\downharpoonright \hspace{-1.111pt}\mathchoice{\hspace{-1.94443pt}}{\hspace{-1.94443pt}}{}{}_{X_i}} \mathchoice{\hspace{-0.55542pt}}{\hspace{-0.55542pt}}{\hspace{-0.27771pt}}{} = u_i$, and $U( s \mathop {\hspace{-1.66656pt}\downharpoonright \hspace{-1.111pt}\mathchoice{\hspace{-1.94443pt}}{\hspace{-1.94443pt}}{}{}_{X_i}} \mathchoice{\hspace{-0.55542pt}}{\hspace{-0.55542pt}}{\hspace{-0.27771pt}}{} ) \in u_i$. $\square$

We now begin preparations for the proof of the second Requirement in the SCD case.

#### Lemma 5

If $\tau \mathop {\hspace{-1.66656pt}\downharpoonright \hspace{-1.111pt}\mathchoice{\hspace{-1.94443pt}}{\hspace{-1.94443pt}}{}{}_{\bar{X}}} \mathchoice{\hspace{-0.55542pt}}{\hspace{-0.55542pt}}{\hspace{-0.27771pt}}{} \subseteq {\mathscr {P}}_{\bar{X}}$, then for every PCSP $(t \rightarrow s) \in \tau$, there exists a path $(a \rightarrow _*t \rightarrow s) \subseteq \tau$ and a subsplit *u* in ${\mathscr {P}}_{\bar{X}}$ such that $a \mathop {\hspace{-1.66656pt}\downharpoonright \hspace{-1.111pt}\mathchoice{\hspace{-1.94443pt}}{\hspace{-1.94443pt}}{}{}_{\bar{X}}} \mathchoice{\hspace{-0.55542pt}}{\hspace{-0.55542pt}}{\hspace{-0.27771pt}}{} = u$ and $U( s \mathop {\hspace{-1.66656pt}\downharpoonright \hspace{-1.111pt}\mathchoice{\hspace{-1.94443pt}}{\hspace{-1.94443pt}}{}{}_{\bar{X}}} \mathchoice{\hspace{-0.55542pt}}{\hspace{-0.55542pt}}{\hspace{-0.27771pt}}{} ) \in u$. If $s \mathop {\hspace{-1.66656pt}\downharpoonright \hspace{-1.111pt}\mathchoice{\hspace{-1.94443pt}}{\hspace{-1.94443pt}}{}{}_{\bar{X}}} \mathchoice{\hspace{-0.55542pt}}{\hspace{-0.55542pt}}{\hspace{-0.27771pt}}{}$ is nontrivial, then $(u \rightarrow s \mathop {\hspace{-1.66656pt}\downharpoonright \hspace{-1.111pt}\mathchoice{\hspace{-1.94443pt}}{\hspace{-1.94443pt}}{}{}_{\bar{X}}} \mathchoice{\hspace{-0.55542pt}}{\hspace{-0.55542pt}}{\hspace{-0.27771pt}}{} ) \in {\mathscr {P}}_{\bar{X}}$.

#### Proof

If $t \mathop {\hspace{-1.66656pt}\downharpoonright \hspace{-1.111pt}\mathchoice{\hspace{-1.94443pt}}{\hspace{-1.94443pt}}{}{}_{\bar{X}}} \mathchoice{\hspace{-0.55542pt}}{\hspace{-0.55542pt}}{\hspace{-0.27771pt}}{}$ is a subsplit in ${\mathscr {P}}_{\bar{X}}$, we know $U( s \mathop {\hspace{-1.66656pt}\downharpoonright \hspace{-1.111pt}\mathchoice{\hspace{-1.94443pt}}{\hspace{-1.94443pt}}{}{}_{\bar{X}}} \mathchoice{\hspace{-0.55542pt}}{\hspace{-0.55542pt}}{\hspace{-0.27771pt}}{} ) \in t \mathop {\hspace{-1.66656pt}\downharpoonright \hspace{-1.111pt}\mathchoice{\hspace{-1.94443pt}}{\hspace{-1.94443pt}}{}{}_{\bar{X}}} \mathchoice{\hspace{-0.55542pt}}{\hspace{-0.55542pt}}{\hspace{-0.27771pt}}{}$, so $u = t \mathop {\hspace{-1.66656pt}\downharpoonright \hspace{-1.111pt}\mathchoice{\hspace{-1.94443pt}}{\hspace{-1.94443pt}}{}{}_{\bar{X}}} \mathchoice{\hspace{-0.55542pt}}{\hspace{-0.55542pt}}{\hspace{-0.27771pt}}{}$, and we are done. If $t \mathop {\hspace{-1.66656pt}\downharpoonright \hspace{-1.111pt}\mathchoice{\hspace{-1.94443pt}}{\hspace{-1.94443pt}}{}{}_{\bar{X}}} \mathchoice{\hspace{-0.55542pt}}{\hspace{-0.55542pt}}{\hspace{-0.27771pt}}{}$ is not a subsplit in ${\mathscr {P}}_{\bar{X}}$, i.e. $t \mathop {\hspace{-1.66656pt}\downharpoonright \hspace{-1.111pt}\mathchoice{\hspace{-1.94443pt}}{\hspace{-1.94443pt}}{}{}_{\bar{X}}} \mathchoice{\hspace{-0.55542pt}}{\hspace{-0.55542pt}}{\hspace{-0.27771pt}}{}$ is trivial, but is not $\pi _{\bar{X}}$ nor a singleton, then we know $U( s \mathop {\hspace{-1.66656pt}\downharpoonright \hspace{-1.111pt}\mathchoice{\hspace{-1.94443pt}}{\hspace{-1.94443pt}}{}{}_{\bar{X}}} \mathchoice{\hspace{-0.55542pt}}{\hspace{-0.55542pt}}{\hspace{-0.27771pt}}{} ) = U( t \mathop {\hspace{-1.66656pt}\downharpoonright \hspace{-1.111pt}\mathchoice{\hspace{-1.94443pt}}{\hspace{-1.94443pt}}{}{}_{\bar{X}}} \mathchoice{\hspace{-0.55542pt}}{\hspace{-0.55542pt}}{\hspace{-0.27771pt}}{} ) \in \pi _{\tau }{(t)} \mathop {\hspace{-1.66656pt}\downharpoonright \hspace{-1.111pt}\mathchoice{\hspace{-1.94443pt}}{\hspace{-1.94443pt}}{}{}_{\bar{X}}} \mathchoice{\hspace{-0.55542pt}}{\hspace{-0.55542pt}}{\hspace{-0.27771pt}}{}$. If $\pi _{\tau }{(t)} \mathop {\hspace{-1.66656pt}\downharpoonright \hspace{-1.111pt}\mathchoice{\hspace{-1.94443pt}}{\hspace{-1.94443pt}}{}{}_{\bar{X}}} \mathchoice{\hspace{-0.55542pt}}{\hspace{-0.55542pt}}{\hspace{-0.27771pt}}{}$ is a subsplit in ${\mathscr {P}}_{\bar{X}}$ then we are done. Otherwise, we can continue this reasoning and chain of equalities, proceeding up the tree until we reach a parent subsplit in ${\mathscr {P}}_{\bar{X}}$ or $\pi _{\bar{X}}$, which we know is a parent subsplit in ${\mathscr {P}}_{\bar{X}}$. If $s \mathop {\hspace{-1.66656pt}\downharpoonright \hspace{-1.111pt}\mathchoice{\hspace{-1.94443pt}}{\hspace{-1.94443pt}}{}{}_{\bar{X}}} \mathchoice{\hspace{-0.55542pt}}{\hspace{-0.55542pt}}{\hspace{-0.27771pt}}{}$ is nontrivial, then there is an uninterrupted path of trivial subsplits between *s* and the *a* we find above. Thus, by tree restriction, $(u \rightarrow s \mathop {\hspace{-1.66656pt}\downharpoonright \hspace{-1.111pt}\mathchoice{\hspace{-1.94443pt}}{\hspace{-1.94443pt}}{}{}_{\bar{X}}} \mathchoice{\hspace{-0.55542pt}}{\hspace{-0.55542pt}}{\hspace{-0.27771pt}}{} )$ is a valid PCSP in ${\mathscr {P}}_{\bar{X}}$. $\square$

#### Lemma 6

Suppose there exists a tree topology $\tau$ with taxon set *X* such that $\tau \mathop {\hspace{-1.66656pt}\downharpoonright \hspace{-1.111pt}\mathchoice{\hspace{-1.94443pt}}{\hspace{-1.94443pt}}{}{}_{X_1}} \mathchoice{\hspace{-0.55542pt}}{\hspace{-0.55542pt}}{\hspace{-0.27771pt}}{} \subseteq {\mathscr {P}}_{X_1}$ and $\tau \mathop {\hspace{-1.66656pt}\downharpoonright \hspace{-1.111pt}\mathchoice{\hspace{-1.94443pt}}{\hspace{-1.94443pt}}{}{}_{X_2}} \mathchoice{\hspace{-0.55542pt}}{\hspace{-0.55542pt}}{\hspace{-0.27771pt}}{} \subseteq {\mathscr {P}}_{X_2}$. If $(t \rightarrow s) \in \tau$ and the algorithm reaches state

$$\begin{aligned} (({t},\underline{U(s)}), ({ a_1 \mathop {\hspace{-1.66656pt}\downharpoonright \hspace{-1.111pt}\mathchoice{\hspace{-1.94443pt}}{\hspace{-1.94443pt}}{}{}_{X_1}} \mathchoice{\hspace{-0.55542pt}}{\hspace{-0.55542pt}}{\hspace{-0.27771pt}}{} },\underline{U(s) \cap X_1}), ({ a_2 \mathop {\hspace{-1.66656pt}\downharpoonright \hspace{-1.111pt}\mathchoice{\hspace{-1.94443pt}}{\hspace{-1.94443pt}}{}{}_{X_2}} \mathchoice{\hspace{-0.55542pt}}{\hspace{-0.55542pt}}{\hspace{-0.27771pt}}{} },\underline{U(s) \cap X_2})), \end{aligned}$$

where $a_1$ is the most recent ancestor of *s* that restricts to a subsplit in ${\mathscr {P}}_{X_1}$, and $a_2$ is most recent ancestor of *s* that restricts to a subsplit in ${\mathscr {P}}_{X_2}$, then $(t \rightarrow s) \in M({\mathscr {P}}_{X_1}, {\mathscr {P}}_{X_2})$.

#### Proof

Similar to Algorithm 1, in general we know

$$\begin{aligned} W_1&:=U(s) \cap X_1 = (Y \cup Z) \cap X_1 \\&= (Y \cap X_1) \cup (Z \cap X_1) = U( s \mathop {\hspace{-1.66656pt}\downharpoonright \hspace{-1.111pt}\mathchoice{\hspace{-1.94443pt}}{\hspace{-1.94443pt}}{}{}_{X_1}} \mathchoice{\hspace{-0.55542pt}}{\hspace{-0.55542pt}}{\hspace{-0.27771pt}}{} ), \end{aligned}$$

and via the same logic $W_2 = U( s \mathop {\hspace{-1.66656pt}\downharpoonright \hspace{-1.111pt}\mathchoice{\hspace{-1.94443pt}}{\hspace{-1.94443pt}}{}{}_{X_2}} \mathchoice{\hspace{-0.55542pt}}{\hspace{-0.55542pt}}{\hspace{-0.27771pt}}{} )$. Let $u_1 = a_1 \mathop {\hspace{-1.66656pt}\downharpoonright \hspace{-1.111pt}\mathchoice{\hspace{-1.94443pt}}{\hspace{-1.94443pt}}{}{}_{X_1}} \mathchoice{\hspace{-0.55542pt}}{\hspace{-0.55542pt}}{\hspace{-0.27771pt}}{}$ and $u_2 = a_2 \mathop {\hspace{-1.66656pt}\downharpoonright \hspace{-1.111pt}\mathchoice{\hspace{-1.94443pt}}{\hspace{-1.94443pt}}{}{}_{X_2}} \mathchoice{\hspace{-0.55542pt}}{\hspace{-0.55542pt}}{\hspace{-0.27771pt}}{}$. By Lemma 5, we know $W_1 \in u_1$ and $W_2 \in u_2$. If $s_1 = s \mathop {\hspace{-1.66656pt}\downharpoonright \hspace{-1.111pt}\mathchoice{\hspace{-1.94443pt}}{\hspace{-1.94443pt}}{}{}_{X_1}} \mathchoice{\hspace{-0.55542pt}}{\hspace{-0.55542pt}}{\hspace{-0.27771pt}}{}$ is trivial, then $U(s_1) = W_1$ and is in ${\mathscr {P}}_{X_1}(({t_1},\underline{W_1}))$ by construction. If $s_1$ is nontrivial, then also by Lemma 5 we know $s_1 \in {\mathscr {P}}_{X_1}(({t_1},\underline{W_1}))$. Similarly, $s_2 = s \mathop {\hspace{-1.66656pt}\downharpoonright \hspace{-1.111pt}\mathchoice{\hspace{-1.94443pt}}{\hspace{-1.94443pt}}{}{}_{X_2}} \mathchoice{\hspace{-0.55542pt}}{\hspace{-0.55542pt}}{\hspace{-0.27771pt}}{} \in {\mathscr {P}}_{X_1}(({t_2},\underline{W_2}))$, so Algorithm 2 considers $s_1$ and $s_2$ at this step. Then one of the subsplits on *X* that the algorithm generates is

$$\begin{aligned} \{[Y \cap X_1] \cup [Y \cap X_2], [Z \cap X_1] \cup [Z \cap X_2]\}&= \{Y \cap [X_1 \cup X_2], Z \cap [X_1 \cup X_2]\} \\&= \{Y \cap X, Z \cap X\} = s. \end{aligned}$$

We know *s* is nontrivial, so $(t \rightarrow s)$ is added to $M({\mathscr {P}}_{X_1}, {\mathscr {P}}_{X_2})$. $\square$

The following theorem establishes the second Requirement in the SCD case.

#### Theorem 7

If there exists a tree topology $\tau$ with taxon set *X* such that $\tau \mathop {\hspace{-1.66656pt}\downharpoonright \hspace{-1.111pt}\mathchoice{\hspace{-1.94443pt}}{\hspace{-1.94443pt}}{}{}_{X_1}} \mathchoice{\hspace{-0.55542pt}}{\hspace{-0.55542pt}}{\hspace{-0.27771pt}}{} \subseteq {\mathscr {P}}_{X_1}$ and $\tau \mathop {\hspace{-1.66656pt}\downharpoonright \hspace{-1.111pt}\mathchoice{\hspace{-1.94443pt}}{\hspace{-1.94443pt}}{}{}_{X_2}} \mathchoice{\hspace{-0.55542pt}}{\hspace{-0.55542pt}}{\hspace{-0.27771pt}}{} \subseteq {\mathscr {P}}_{X_2}$, then $\tau \subseteq M({\mathscr {P}}_{X_1}, {\mathscr {P}}_{X_2})$.

#### Proof

We proceed via recursive proof by induction over all PCSPs $(t \rightarrow s) \in \tau$, with $s = \{Y, Z\}$. Our base case is the algorithm’s first state $({t},\underline{W}) = ({\pi _{X}},\underline{X})$, $t_1 = \pi _{X_1}$, and $t_2 = \pi _{X_2}$.

We know *s* only has one ancestor, $\pi _{X}$, which restricts to $\pi _{X_1}$ in ${\mathscr {P}}_{X_1}$ and to $\pi _{X_2}$ in ${\mathscr {P}}_{X_2}$. This satisfies the criteria of Lemma 6, so $(t \rightarrow s)$ will be in $M({\mathscr {P}}_{X_1}, {\mathscr {P}}_{X_2})$.

Our inductive step for general $(t \rightarrow s) \in \tau$ uses the same argument, but uses the inductive assumption that PCSP $(\pi _{\tau }{(t)} \rightarrow t)$ was previously added to the output. Given this assumption, we will show that the algorithm constructs the state triplet

$$\begin{aligned} (({t},\underline{U(s)}), ({ a_1 \mathop {\hspace{-1.66656pt}\downharpoonright \hspace{-1.111pt}\mathchoice{\hspace{-1.94443pt}}{\hspace{-1.94443pt}}{}{}_{X_1}} \mathchoice{\hspace{-0.55542pt}}{\hspace{-0.55542pt}}{\hspace{-0.27771pt}}{} },\underline{U(s) \cap X_1}), ({ a_2 \mathop {\hspace{-1.66656pt}\downharpoonright \hspace{-1.111pt}\mathchoice{\hspace{-1.94443pt}}{\hspace{-1.94443pt}}{}{}_{X_2}} \mathchoice{\hspace{-0.55542pt}}{\hspace{-0.55542pt}}{\hspace{-0.27771pt}}{} },\underline{U(s) \cap X_2})). \end{aligned}$$

If the algorithm reaches this state, then Lemma 6 guarantees that $(t \rightarrow s)$ will be in $M({\mathscr {P}}_{X_1}, {\mathscr {P}}_{X_2})$. Lemma 5 guarantees that such $a_1 \mathop {\hspace{-1.66656pt}\downharpoonright \hspace{-1.111pt}\mathchoice{\hspace{-1.94443pt}}{\hspace{-1.94443pt}}{}{}_{X_1}} \mathchoice{\hspace{-0.55542pt}}{\hspace{-0.55542pt}}{\hspace{-0.27771pt}}{}$ and $a_2 \mathop {\hspace{-1.66656pt}\downharpoonright \hspace{-1.111pt}\mathchoice{\hspace{-1.94443pt}}{\hspace{-1.94443pt}}{}{}_{X_2}} \mathchoice{\hspace{-0.55542pt}}{\hspace{-0.55542pt}}{\hspace{-0.27771pt}}{}$ exist.

Since $(\pi _{\tau }{(t)} \rightarrow t)$ has already been visited and $|U(s) |> 2$, we know that there is a triplet in the stack with $({t},\underline{U(s)})$ as the first component. Considering the other components of this triplet, if *t* restricts to a subsplit in ${\mathscr {P}}_{X_1}$, then $a_1 = t$ and $u_1 = t \mathop {\hspace{-1.66656pt}\downharpoonright \hspace{-1.111pt}\mathchoice{\hspace{-1.94443pt}}{\hspace{-1.94443pt}}{}{}_{X_1}} \mathchoice{\hspace{-0.55542pt}}{\hspace{-0.55542pt}}{\hspace{-0.27771pt}}{}$. If not, then *t* and *s* have the same most recent ancestor that restricts to a subsplit in ${\mathscr {P}}_{X_1}$, so the algorithm passes it along as $u_1$. Either way, $({ a_1 \mathop {\hspace{-1.66656pt}\downharpoonright \hspace{-1.111pt}\mathchoice{\hspace{-1.94443pt}}{\hspace{-1.94443pt}}{}{}_{X_1}} \mathchoice{\hspace{-0.55542pt}}{\hspace{-0.55542pt}}{\hspace{-0.27771pt}}{} },\underline{U(s) \cap X_1})$ is the second component. The same argument holds for $X_2$ and the third component. This is exactly the state triplet we require, so by Lemma 6, we know $(t \rightarrow s) \in M({\mathscr {P}}_{X_1}, {\mathscr {P}}_{X_2})$. Therefore, by induction, all $(t \rightarrow s) \in \tau$ are in $M({\mathscr {P}}_{X_1}, {\mathscr {P}}_{X_2})$, so $\tau \subseteq M({\mathscr {P}}_{X_1}, {\mathscr {P}}_{X_2})$. $\square$

Finally, we show that Algorithm 2 runs in $O(n_{P_1}, n_{P_2})$ time, where $n_{P_i}$ is the number of PCSPs in reference support ${\mathscr {P}}_{X_i}$. We know Algorithm 2 only visits each subsplit and clade triplet $(({t},\underline{W}), ({t_1},\underline{W_1}), ({t_2},\underline{W_2}))$ at most once.

Next, we show that for each pair $({t_1},\underline{W_1}), ({t_2},\underline{W_2})$ in the second and third components, there are at most three $({t},\underline{W})$ that can fill the first component. If $({t_1},\underline{W_1}) = {W'_1}/W_1$ and $({t_2},\underline{W_2}) = {W'_2}/W_2$, then candidates for $({t},\underline{W})$ are,

$$\begin{aligned} (W'_1 \cup W'_2){} & {} / (W_1 \cup W_2), \\ W'_1{} & {} / (W_1 \cup W_2), \\ W'_2{} & {} / (W_1 \cup W_2). \end{aligned}$$

The first candidate is the natural mutual subsplit and clade that restricts to $({t_1},\underline{W_1}), ({t_2},\underline{W_2})$. The latter two candidates occur only when $({t},\underline{W})$ is trivial on restriction to $X_i$, passing $({t_i},\underline{W_i})$ back to the stack. Since the only trivial descendant of $W'_i / W_i$ is $\emptyset / W_i$, we get the latter two candidates by combining $\emptyset / W_i$ with the other subsplit and clade. There is no fourth candidate because combining $\emptyset / W_1$ with $\emptyset / W_2$ results in the trivial subsplit and clade $\emptyset / W_1 \cup W_2$.

So in the worst case of disjoint $X_1, X_2$, where any $({t_1},\underline{W_1})$ can be paired with any $({t_2},\underline{W_2})$, every PCSP in ${\mathscr {P}}_{X_1}$ might have to be crossed with every PCSP in ${\mathscr {P}}_{X_2}$ at most three times. Because we keep track of the subsplit-clade pairs that have already been pushed onto the stack to prevent them being pushed multiple times, the algorithm runs in $O(n_{P_1} n_{P_2})$ time.

### CCD Gradient Derivations

For subsplits *s* and $s'$, a standard derivative result for softmax parameters gives us,

A1 $$\begin{aligned} \partial _{s'} q(s \mid U(s)) = q(s' \mid U(s')) \left[ 1_{\{s=s'\}} - q(s \mid U(s')) \right] , \end{aligned}$$

if $U(s) = U(s')$ and zero otherwise. If we have CCD-parameterized SBNs *p* and *q* on tip sets $\bar{X}$ and *X*, we can use Eq. 2 to take the derivative of the KL-divergence between *p* and *q* restricted to $\bar{X}$,

A2 $$\begin{aligned} \partial _{s'} D_{\text {KL}}(p \parallel q \mathop {\hspace{-1.66656pt}\downharpoonright \hspace{-1.111pt}\mathchoice{\hspace{-1.94443pt}}{\hspace{-1.94443pt}}{}{}_{\bar{X}}} \mathchoice{\hspace{-0.55542pt}}{\hspace{-0.55542pt}}{\hspace{-0.27771pt}}{} )&= -\sum _{\bar{s}} p(\bar{s}) \, \partial _{s'} \log q \mathop {\hspace{-1.66656pt}\downharpoonright \hspace{-1.111pt}\mathchoice{\hspace{-1.94443pt}}{\hspace{-1.94443pt}}{}{}_{\bar{X}}} \mathchoice{\hspace{-0.55542pt}}{\hspace{-0.55542pt}}{\hspace{-0.27771pt}}{} (\bar{s} \vert U(\bar{s})) \nonumber \\ {}&= -\sum _{\bar{s}} \frac{p(\bar{s})}{ q \mathop {\hspace{-1.66656pt}\downharpoonright \hspace{-1.111pt}\mathchoice{\hspace{-1.94443pt}}{\hspace{-1.94443pt}}{}{}_{\bar{X}}} \mathchoice{\hspace{-0.55542pt}}{\hspace{-0.55542pt}}{\hspace{-0.27771pt}}{} (\bar{s} \vert U(\bar{s}))} \, \partial _{s'} \, q \mathop {\hspace{-1.66656pt}\downharpoonright \hspace{-1.111pt}\mathchoice{\hspace{-1.94443pt}}{\hspace{-1.94443pt}}{}{}_{\bar{X}}} \mathchoice{\hspace{-0.55542pt}}{\hspace{-0.55542pt}}{\hspace{-0.27771pt}}{} (\bar{s} \vert U(\bar{s})). \end{aligned}$$

We can then use Eqs. 4, 5, and 6 to break the formula down to solely depend on the derivative of the unconditional subsplit probability. For subsplit $\bar{s}$ and clade $\bar{U}$ in $\bar{X}$,

A3 $$\begin{aligned} \partial _{s'} \, q \mathop {\hspace{-1.66656pt}\downharpoonright \hspace{-1.111pt}\mathchoice{\hspace{-1.94443pt}}{\hspace{-1.94443pt}}{}{}_{\bar{X}}} \mathchoice{\hspace{-0.55542pt}}{\hspace{-0.55542pt}}{\hspace{-0.27771pt}}{} (\bar{s} \vert U(\bar{s}))&= \frac{ q \mathop {\hspace{-1.66656pt}\downharpoonright \hspace{-1.111pt}\mathchoice{\hspace{-1.94443pt}}{\hspace{-1.94443pt}}{}{}_{\bar{X}}} \mathchoice{\hspace{-0.55542pt}}{\hspace{-0.55542pt}}{\hspace{-0.27771pt}}{} (U(\bar{s})) \cdot \partial _{s'} q \mathop {\hspace{-1.66656pt}\downharpoonright \hspace{-1.111pt}\mathchoice{\hspace{-1.94443pt}}{\hspace{-1.94443pt}}{}{}_{\bar{X}}} \mathchoice{\hspace{-0.55542pt}}{\hspace{-0.55542pt}}{\hspace{-0.27771pt}}{} (\bar{s}) - q \mathop {\hspace{-1.66656pt}\downharpoonright \hspace{-1.111pt}\mathchoice{\hspace{-1.94443pt}}{\hspace{-1.94443pt}}{}{}_{\bar{X}}} \mathchoice{\hspace{-0.55542pt}}{\hspace{-0.55542pt}}{\hspace{-0.27771pt}}{} (\bar{s}) \cdot \partial _{s'} q \mathop {\hspace{-1.66656pt}\downharpoonright \hspace{-1.111pt}\mathchoice{\hspace{-1.94443pt}}{\hspace{-1.94443pt}}{}{}_{\bar{X}}} \mathchoice{\hspace{-0.55542pt}}{\hspace{-0.55542pt}}{\hspace{-0.27771pt}}{} (U(\bar{s})) }{ q \mathop {\hspace{-1.66656pt}\downharpoonright \hspace{-1.111pt}\mathchoice{\hspace{-1.94443pt}}{\hspace{-1.94443pt}}{}{}_{\bar{X}}} \mathchoice{\hspace{-0.55542pt}}{\hspace{-0.55542pt}}{\hspace{-0.27771pt}}{} (U(\bar{s}))^{2} } \nonumber \\&= \frac{ \partial _{s'} q \mathop {\hspace{-1.66656pt}\downharpoonright \hspace{-1.111pt}\mathchoice{\hspace{-1.94443pt}}{\hspace{-1.94443pt}}{}{}_{\bar{X}}} \mathchoice{\hspace{-0.55542pt}}{\hspace{-0.55542pt}}{\hspace{-0.27771pt}}{} (\bar{s}) - q \mathop {\hspace{-1.66656pt}\downharpoonright \hspace{-1.111pt}\mathchoice{\hspace{-1.94443pt}}{\hspace{-1.94443pt}}{}{}_{\bar{X}}} \mathchoice{\hspace{-0.55542pt}}{\hspace{-0.55542pt}}{\hspace{-0.27771pt}}{} (\bar{s} \vert U(\bar{s})) \cdot \partial _{s'} q \mathop {\hspace{-1.66656pt}\downharpoonright \hspace{-1.111pt}\mathchoice{\hspace{-1.94443pt}}{\hspace{-1.94443pt}}{}{}_{\bar{X}}} \mathchoice{\hspace{-0.55542pt}}{\hspace{-0.55542pt}}{\hspace{-0.27771pt}}{} (U(\bar{s})) }{ q \mathop {\hspace{-1.66656pt}\downharpoonright \hspace{-1.111pt}\mathchoice{\hspace{-1.94443pt}}{\hspace{-1.94443pt}}{}{}_{\bar{X}}} \mathchoice{\hspace{-0.55542pt}}{\hspace{-0.55542pt}}{\hspace{-0.27771pt}}{} (U(\bar{s})) }, \nonumber \\ \partial _{s'} q \mathop {\hspace{-1.66656pt}\downharpoonright \hspace{-1.111pt}\mathchoice{\hspace{-1.94443pt}}{\hspace{-1.94443pt}}{}{}_{\bar{X}}} \mathchoice{\hspace{-0.55542pt}}{\hspace{-0.55542pt}}{\hspace{-0.27771pt}}{} (\bar{s})&= \sum _{ s \mathop {\hspace{-1.66656pt}\downharpoonright \hspace{-1.111pt}\mathchoice{\hspace{-1.94443pt}}{\hspace{-1.94443pt}}{}{}_{\bar{X}}} \mathchoice{\hspace{-0.55542pt}}{\hspace{-0.55542pt}}{\hspace{-0.27771pt}}{} = \bar{s}} \partial _{s'} q(s),\nonumber \\ \partial _{s'} q \mathop {\hspace{-1.66656pt}\downharpoonright \hspace{-1.111pt}\mathchoice{\hspace{-1.94443pt}}{\hspace{-1.94443pt}}{}{}_{\bar{X}}} \mathchoice{\hspace{-0.55542pt}}{\hspace{-0.55542pt}}{\hspace{-0.27771pt}}{} (\bar{U})&= \sum _{\bar{s}:U(\bar{s})=\bar{U}} \partial _{s'} q \mathop {\hspace{-1.66656pt}\downharpoonright \hspace{-1.111pt}\mathchoice{\hspace{-1.94443pt}}{\hspace{-1.94443pt}}{}{}_{\bar{X}}} \mathchoice{\hspace{-0.55542pt}}{\hspace{-0.55542pt}}{\hspace{-0.27771pt}}{} (\bar{s}) = \sum _{\bar{s}:U(\bar{s})=\bar{U}} \sum _{ s \mathop {\hspace{-1.66656pt}\downharpoonright \hspace{-1.111pt}\mathchoice{\hspace{-1.94443pt}}{\hspace{-1.94443pt}}{}{}_{\bar{X}}} \mathchoice{\hspace{-0.55542pt}}{\hspace{-0.55542pt}}{\hspace{-0.27771pt}}{} = \bar{s}} \partial _{s'} q(s). \end{aligned}$$

Thus all of our derivatives depend on the derivative of the unconditional subsplit probability. If we are taking a derivative with respect to $v_{s'}$, we can then use the law of total probability and SBN conditional independence to split our unconditional subsplit probabilities into the collection of paths that pass through $U(s')$ and the paths that do not. We use the notation $C_{s'} :=q(U(s') \notin \{\pi _{X} \rightarrow _*s\})$ to capture the paths that do not pass through $U(s')$ and will therefore be a constant with respect to $v_{s'}$.

$$\begin{aligned} q(s)&= q(U(s') \rightarrow _*s) + q(U(s') \notin \{\pi _{X} \rightarrow _*s\}) \\&= \sum _{s'':U(s') \rightarrow s''} q(U(s') \rightarrow s'' \rightarrow _*s) + C_{s'} \\&= q(U(s')) \sum _{s'':U(s') \rightarrow s''} q(s'' \vert U(s')) \, q(s'' \rightarrow _*s \vert s'') + C_{s'}. \end{aligned}$$

Finally, we can use Eq. A1 to express the unconditional probability derivative in terms of conditional and unconditional probabilities, readily available from the SBN itself.

A4 $$\begin{aligned}&\partial _{s'} q(s) \nonumber \\&= q(U(s')) \sum _{s'':U(s') \rightarrow s''} \partial _{s' \vert U(s')} \, q(s'' \vert U(s')) \, q(s'' \rightarrow _*s \mid s'')\nonumber \\&= q(U(s')) \sum _{s'':U(s') \rightarrow s''} q(s' \vert U(s')) \left[ 1_{\{s''=s'\}} - q(s'' \vert U(s')) \right] q(s'' \rightarrow _*s \mid s'') \nonumber \\&= q(U(s')) q(s' \vert U(s')) \left[ q(s' \rightarrow _*s \mid s') - \sum _{s'':U(s') \rightarrow s''} q(s'' \vert U(s')) q(s'' \rightarrow _*s \mid s'') \right] \nonumber \\&= q(U(s')) q(s' \vert U(s')) \left[ q(s' \rightarrow _*s \mid s') - q(U(s') \rightarrow _*s \mid U(s')). \right] \end{aligned}$$

Combining Eqs. A2, A3, and 5 gives us a relatively succinct formula for the derivative,

$$\begin{aligned} \partial _{s'} D_{\text {KL}}(p \parallel q \mathop {\hspace{-1.66656pt}\downharpoonright \hspace{-1.111pt}\mathchoice{\hspace{-1.94443pt}}{\hspace{-1.94443pt}}{}{}_{\bar{X}}} \mathchoice{\hspace{-0.55542pt}}{\hspace{-0.55542pt}}{\hspace{-0.27771pt}}{} )&= -\sum _{\bar{s}} \frac{p(\bar{s})}{ q \mathop {\hspace{-1.66656pt}\downharpoonright \hspace{-1.111pt}\mathchoice{\hspace{-1.94443pt}}{\hspace{-1.94443pt}}{}{}_{\bar{X}}} \mathchoice{\hspace{-0.55542pt}}{\hspace{-0.55542pt}}{\hspace{-0.27771pt}}{} (\bar{s} \vert U(\bar{s}))} \, \partial _{s'} \, q \mathop {\hspace{-1.66656pt}\downharpoonright \hspace{-1.111pt}\mathchoice{\hspace{-1.94443pt}}{\hspace{-1.94443pt}}{}{}_{\bar{X}}} \mathchoice{\hspace{-0.55542pt}}{\hspace{-0.55542pt}}{\hspace{-0.27771pt}}{} (\bar{s} \vert U(\bar{s})) \\&= -\sum _{\bar{s}} \frac{ p(\bar{s}) }{ q \mathop {\hspace{-1.66656pt}\downharpoonright \hspace{-1.111pt}\mathchoice{\hspace{-1.94443pt}}{\hspace{-1.94443pt}}{}{}_{\bar{X}}} \mathchoice{\hspace{-0.55542pt}}{\hspace{-0.55542pt}}{\hspace{-0.27771pt}}{} (\bar{s}) } \left[ \partial _{s'} q \mathop {\hspace{-1.66656pt}\downharpoonright \hspace{-1.111pt}\mathchoice{\hspace{-1.94443pt}}{\hspace{-1.94443pt}}{}{}_{\bar{X}}} \mathchoice{\hspace{-0.55542pt}}{\hspace{-0.55542pt}}{\hspace{-0.27771pt}}{} (\bar{s}) - q \mathop {\hspace{-1.66656pt}\downharpoonright \hspace{-1.111pt}\mathchoice{\hspace{-1.94443pt}}{\hspace{-1.94443pt}}{}{}_{\bar{X}}} \mathchoice{\hspace{-0.55542pt}}{\hspace{-0.55542pt}}{\hspace{-0.27771pt}}{} (\bar{s} \vert U(\bar{s})) \cdot \partial _{s'} q \mathop {\hspace{-1.66656pt}\downharpoonright \hspace{-1.111pt}\mathchoice{\hspace{-1.94443pt}}{\hspace{-1.94443pt}}{}{}_{\bar{X}}} \mathchoice{\hspace{-0.55542pt}}{\hspace{-0.55542pt}}{\hspace{-0.27771pt}}{} (U(\bar{s})) \right] \\&= -\sum _{\bar{s}} \frac{ p(\bar{s}) }{ q \mathop {\hspace{-1.66656pt}\downharpoonright \hspace{-1.111pt}\mathchoice{\hspace{-1.94443pt}}{\hspace{-1.94443pt}}{}{}_{\bar{X}}} \mathchoice{\hspace{-0.55542pt}}{\hspace{-0.55542pt}}{\hspace{-0.27771pt}}{} (\bar{s}) } \partial _{s'} q \mathop {\hspace{-1.66656pt}\downharpoonright \hspace{-1.111pt}\mathchoice{\hspace{-1.94443pt}}{\hspace{-1.94443pt}}{}{}_{\bar{X}}} \mathchoice{\hspace{-0.55542pt}}{\hspace{-0.55542pt}}{\hspace{-0.27771pt}}{} (\bar{s}) + \sum _{\bar{s}} \frac{ \partial _{s'} q \mathop {\hspace{-1.66656pt}\downharpoonright \hspace{-1.111pt}\mathchoice{\hspace{-1.94443pt}}{\hspace{-1.94443pt}}{}{}_{\bar{X}}} \mathchoice{\hspace{-0.55542pt}}{\hspace{-0.55542pt}}{\hspace{-0.27771pt}}{} (U(\bar{s})) }{ q \mathop {\hspace{-1.66656pt}\downharpoonright \hspace{-1.111pt}\mathchoice{\hspace{-1.94443pt}}{\hspace{-1.94443pt}}{}{}_{\bar{X}}} \mathchoice{\hspace{-0.55542pt}}{\hspace{-0.55542pt}}{\hspace{-0.27771pt}}{} (U(\bar{s})) } p(\bar{s}) \\&= \sum _{\bar{U}} \frac{ \partial _{s'} q \mathop {\hspace{-1.66656pt}\downharpoonright \hspace{-1.111pt}\mathchoice{\hspace{-1.94443pt}}{\hspace{-1.94443pt}}{}{}_{\bar{X}}} \mathchoice{\hspace{-0.55542pt}}{\hspace{-0.55542pt}}{\hspace{-0.27771pt}}{} (\bar{U}) }{ q \mathop {\hspace{-1.66656pt}\downharpoonright \hspace{-1.111pt}\mathchoice{\hspace{-1.94443pt}}{\hspace{-1.94443pt}}{}{}_{\bar{X}}} \mathchoice{\hspace{-0.55542pt}}{\hspace{-0.55542pt}}{\hspace{-0.27771pt}}{} (\bar{U}) } \sum _{\bar{s}:U(\bar{s})=\bar{U}} p(\bar{s}) - \sum _{\bar{s}} \frac{ p(\bar{s}) }{ q \mathop {\hspace{-1.66656pt}\downharpoonright \hspace{-1.111pt}\mathchoice{\hspace{-1.94443pt}}{\hspace{-1.94443pt}}{}{}_{\bar{X}}} \mathchoice{\hspace{-0.55542pt}}{\hspace{-0.55542pt}}{\hspace{-0.27771pt}}{} (\bar{s}) } \partial _{s'} q \mathop {\hspace{-1.66656pt}\downharpoonright \hspace{-1.111pt}\mathchoice{\hspace{-1.94443pt}}{\hspace{-1.94443pt}}{}{}_{\bar{X}}} \mathchoice{\hspace{-0.55542pt}}{\hspace{-0.55542pt}}{\hspace{-0.27771pt}}{} (\bar{s}) \\&= \sum _{\bar{U}} \frac{ p(\bar{U}) }{ q \mathop {\hspace{-1.66656pt}\downharpoonright \hspace{-1.111pt}\mathchoice{\hspace{-1.94443pt}}{\hspace{-1.94443pt}}{}{}_{\bar{X}}} \mathchoice{\hspace{-0.55542pt}}{\hspace{-0.55542pt}}{\hspace{-0.27771pt}}{} (\bar{U}) } \partial _{s'} q \mathop {\hspace{-1.66656pt}\downharpoonright \hspace{-1.111pt}\mathchoice{\hspace{-1.94443pt}}{\hspace{-1.94443pt}}{}{}_{\bar{X}}} \mathchoice{\hspace{-0.55542pt}}{\hspace{-0.55542pt}}{\hspace{-0.27771pt}}{} (\bar{U}) - \sum _{\bar{s}} \frac{ p(\bar{s}) }{ q \mathop {\hspace{-1.66656pt}\downharpoonright \hspace{-1.111pt}\mathchoice{\hspace{-1.94443pt}}{\hspace{-1.94443pt}}{}{}_{\bar{X}}} \mathchoice{\hspace{-0.55542pt}}{\hspace{-0.55542pt}}{\hspace{-0.27771pt}}{} (\bar{s}) } \partial _{s'} q \mathop {\hspace{-1.66656pt}\downharpoonright \hspace{-1.111pt}\mathchoice{\hspace{-1.94443pt}}{\hspace{-1.94443pt}}{}{}_{\bar{X}}} \mathchoice{\hspace{-0.55542pt}}{\hspace{-0.55542pt}}{\hspace{-0.27771pt}}{} (\bar{s}). \end{aligned}$$

This form displays one of the derivative’s natural symmetries between clade and subsplit probabilities and derivatives. However, if implemented naively, this form may result in iterating over the subsplit support multiple times. We address this by exchanging summations,

$$\begin{aligned} \partial _{s'} D_{\text {KL}}(p \parallel q \mathop {\hspace{-1.66656pt}\downharpoonright \hspace{-1.111pt}\mathchoice{\hspace{-1.94443pt}}{\hspace{-1.94443pt}}{}{}_{\bar{X}}} \mathchoice{\hspace{-0.55542pt}}{\hspace{-0.55542pt}}{\hspace{-0.27771pt}}{} )&= \sum _{\bar{U}} \frac{ p(\bar{U}) }{ q \mathop {\hspace{-1.66656pt}\downharpoonright \hspace{-1.111pt}\mathchoice{\hspace{-1.94443pt}}{\hspace{-1.94443pt}}{}{}_{\bar{X}}} \mathchoice{\hspace{-0.55542pt}}{\hspace{-0.55542pt}}{\hspace{-0.27771pt}}{} (\bar{U}) } \sum _s \partial _{s'} q(s) 1_{\{U( s \mathop {\hspace{-1.66656pt}\downharpoonright \hspace{-1.111pt}\mathchoice{\hspace{-1.94443pt}}{\hspace{-1.94443pt}}{}{}_{\bar{X}}} \mathchoice{\hspace{-0.55542pt}}{\hspace{-0.55542pt}}{\hspace{-0.27771pt}}{} ) = \bar{U}\}} \\ {}&\quad - \sum _{\bar{s}} \frac{ p(\bar{s}) }{ q \mathop {\hspace{-1.66656pt}\downharpoonright \hspace{-1.111pt}\mathchoice{\hspace{-1.94443pt}}{\hspace{-1.94443pt}}{}{}_{\bar{X}}} \mathchoice{\hspace{-0.55542pt}}{\hspace{-0.55542pt}}{\hspace{-0.27771pt}}{} (\bar{s}) } \sum _s \partial _{s'} q(s) 1_{\{ s \mathop {\hspace{-1.66656pt}\downharpoonright \hspace{-1.111pt}\mathchoice{\hspace{-1.94443pt}}{\hspace{-1.94443pt}}{}{}_{\bar{X}}} \mathchoice{\hspace{-0.55542pt}}{\hspace{-0.55542pt}}{\hspace{-0.27771pt}}{} = \bar{s} \}} \\ {}&= \sum _s \left[ \frac{ p(U( s \mathop {\hspace{-1.66656pt}\downharpoonright \hspace{-1.111pt}\mathchoice{\hspace{-1.94443pt}}{\hspace{-1.94443pt}}{}{}_{\bar{X}}} \mathchoice{\hspace{-0.55542pt}}{\hspace{-0.55542pt}}{\hspace{-0.27771pt}}{} )) }{ q \mathop {\hspace{-1.66656pt}\downharpoonright \hspace{-1.111pt}\mathchoice{\hspace{-1.94443pt}}{\hspace{-1.94443pt}}{}{}_{\bar{X}}} \mathchoice{\hspace{-0.55542pt}}{\hspace{-0.55542pt}}{\hspace{-0.27771pt}}{} (U( s \mathop {\hspace{-1.66656pt}\downharpoonright \hspace{-1.111pt}\mathchoice{\hspace{-1.94443pt}}{\hspace{-1.94443pt}}{}{}_{\bar{X}}} \mathchoice{\hspace{-0.55542pt}}{\hspace{-0.55542pt}}{\hspace{-0.27771pt}}{} )) } - \frac{ p( s \mathop {\hspace{-1.66656pt}\downharpoonright \hspace{-1.111pt}\mathchoice{\hspace{-1.94443pt}}{\hspace{-1.94443pt}}{}{}_{\bar{X}}} \mathchoice{\hspace{-0.55542pt}}{\hspace{-0.55542pt}}{\hspace{-0.27771pt}}{} ) }{ q \mathop {\hspace{-1.66656pt}\downharpoonright \hspace{-1.111pt}\mathchoice{\hspace{-1.94443pt}}{\hspace{-1.94443pt}}{}{}_{\bar{X}}} \mathchoice{\hspace{-0.55542pt}}{\hspace{-0.55542pt}}{\hspace{-0.27771pt}}{} ( s \mathop {\hspace{-1.66656pt}\downharpoonright \hspace{-1.111pt}\mathchoice{\hspace{-1.94443pt}}{\hspace{-1.94443pt}}{}{}_{\bar{X}}} \mathchoice{\hspace{-0.55542pt}}{\hspace{-0.55542pt}}{\hspace{-0.27771pt}}{} ) } \right] \partial _{s'} q(s). \end{aligned}$$

This form clearly shows the algorithmic complexity of the gradient computation as $O({n_{s}}^2)$ where ${n_{s}}$ is the number of subsplits in the support, since both the summation and the derivative traverse every subsplit.

### SCD gradient derivations

For subsplits *s*, $s'$, *t*, and $t'$, our softmax derivative result is then,

A5 $$\begin{aligned} \partial _{s' \vert t'} q(s \vert t) = q(s' \vert ({t'},\underline{U(s)})) \left[ 1_{\{s=s'\}} - q(s \vert ({t'},\underline{U(s)})) \right] , \end{aligned}$$

if $t = t'$ and zero otherwise. The derivative of the KL-divergence is,

A6 $$\begin{aligned} \partial _{s' \vert t'} D_{\text {KL}}(p \parallel q \mathop {\hspace{-1.66656pt}\downharpoonright \hspace{-1.111pt}\mathchoice{\hspace{-1.94443pt}}{\hspace{-1.94443pt}}{}{}_{\bar{X}}} \mathchoice{\hspace{-0.55542pt}}{\hspace{-0.55542pt}}{\hspace{-0.27771pt}}{} )&= -\sum _{(\bar{t} \rightarrow \bar{s})} p(\bar{t} \rightarrow \bar{s}) \, \partial _{s' \vert t'} \log q \mathop {\hspace{-1.66656pt}\downharpoonright \hspace{-1.111pt}\mathchoice{\hspace{-1.94443pt}}{\hspace{-1.94443pt}}{}{}_{\bar{X}}} \mathchoice{\hspace{-0.55542pt}}{\hspace{-0.55542pt}}{\hspace{-0.27771pt}}{} (\bar{s} \vert \bar{t}) \nonumber \\&= -\sum _{(\bar{t} \rightarrow \bar{s})} \frac{p(\bar{t} \rightarrow \bar{s})}{ q \mathop {\hspace{-1.66656pt}\downharpoonright \hspace{-1.111pt}\mathchoice{\hspace{-1.94443pt}}{\hspace{-1.94443pt}}{}{}_{\bar{X}}} \mathchoice{\hspace{-0.55542pt}}{\hspace{-0.55542pt}}{\hspace{-0.27771pt}}{} (\bar{s} \vert \bar{t})} \, \partial _{s' \vert t'} \, q \mathop {\hspace{-1.66656pt}\downharpoonright \hspace{-1.111pt}\mathchoice{\hspace{-1.94443pt}}{\hspace{-1.94443pt}}{}{}_{\bar{X}}} \mathchoice{\hspace{-0.55542pt}}{\hspace{-0.55542pt}}{\hspace{-0.27771pt}}{} (\bar{s} \vert \bar{t}). \end{aligned}$$

For PCSP $(\bar{t} \rightarrow \bar{s})$ we see,

A7 $$\begin{aligned} \partial _{s' \vert t'} \, q \mathop {\hspace{-1.66656pt}\downharpoonright \hspace{-1.111pt}\mathchoice{\hspace{-1.94443pt}}{\hspace{-1.94443pt}}{}{}_{\bar{X}}} \mathchoice{\hspace{-0.55542pt}}{\hspace{-0.55542pt}}{\hspace{-0.27771pt}}{} (\bar{s} \vert \bar{t})&= \frac{ q \mathop {\hspace{-1.66656pt}\downharpoonright \hspace{-1.111pt}\mathchoice{\hspace{-1.94443pt}}{\hspace{-1.94443pt}}{}{}_{\bar{X}}} \mathchoice{\hspace{-0.55542pt}}{\hspace{-0.55542pt}}{\hspace{-0.27771pt}}{} (\bar{t}) \cdot \partial _{s' \vert t'} q \mathop {\hspace{-1.66656pt}\downharpoonright \hspace{-1.111pt}\mathchoice{\hspace{-1.94443pt}}{\hspace{-1.94443pt}}{}{}_{\bar{X}}} \mathchoice{\hspace{-0.55542pt}}{\hspace{-0.55542pt}}{\hspace{-0.27771pt}}{} (\bar{t} \rightarrow \bar{s}) - q \mathop {\hspace{-1.66656pt}\downharpoonright \hspace{-1.111pt}\mathchoice{\hspace{-1.94443pt}}{\hspace{-1.94443pt}}{}{}_{\bar{X}}} \mathchoice{\hspace{-0.55542pt}}{\hspace{-0.55542pt}}{\hspace{-0.27771pt}}{} (\bar{t} \rightarrow \bar{s}) \cdot \partial _{s' \vert t'} q \mathop {\hspace{-1.66656pt}\downharpoonright \hspace{-1.111pt}\mathchoice{\hspace{-1.94443pt}}{\hspace{-1.94443pt}}{}{}_{\bar{X}}} \mathchoice{\hspace{-0.55542pt}}{\hspace{-0.55542pt}}{\hspace{-0.27771pt}}{} (\bar{t}) }{ q \mathop {\hspace{-1.66656pt}\downharpoonright \hspace{-1.111pt}\mathchoice{\hspace{-1.94443pt}}{\hspace{-1.94443pt}}{}{}_{\bar{X}}} \mathchoice{\hspace{-0.55542pt}}{\hspace{-0.55542pt}}{\hspace{-0.27771pt}}{} (\bar{t})^{2} } \nonumber \\&= \frac{ \partial _{s' \vert t'} q \mathop {\hspace{-1.66656pt}\downharpoonright \hspace{-1.111pt}\mathchoice{\hspace{-1.94443pt}}{\hspace{-1.94443pt}}{}{}_{\bar{X}}} \mathchoice{\hspace{-0.55542pt}}{\hspace{-0.55542pt}}{\hspace{-0.27771pt}}{} (\bar{t} \rightarrow \bar{s}) - q \mathop {\hspace{-1.66656pt}\downharpoonright \hspace{-1.111pt}\mathchoice{\hspace{-1.94443pt}}{\hspace{-1.94443pt}}{}{}_{\bar{X}}} \mathchoice{\hspace{-0.55542pt}}{\hspace{-0.55542pt}}{\hspace{-0.27771pt}}{} (\bar{s} \vert \bar{t}) \cdot \partial _{s' \vert t'} q \mathop {\hspace{-1.66656pt}\downharpoonright \hspace{-1.111pt}\mathchoice{\hspace{-1.94443pt}}{\hspace{-1.94443pt}}{}{}_{\bar{X}}} \mathchoice{\hspace{-0.55542pt}}{\hspace{-0.55542pt}}{\hspace{-0.27771pt}}{} (\bar{t}) }{ q \mathop {\hspace{-1.66656pt}\downharpoonright \hspace{-1.111pt}\mathchoice{\hspace{-1.94443pt}}{\hspace{-1.94443pt}}{}{}_{\bar{X}}} \mathchoice{\hspace{-0.55542pt}}{\hspace{-0.55542pt}}{\hspace{-0.27771pt}}{} (\bar{t}) }, \nonumber \\ \partial _{s' \vert t'} q \mathop {\hspace{-1.66656pt}\downharpoonright \hspace{-1.111pt}\mathchoice{\hspace{-1.94443pt}}{\hspace{-1.94443pt}}{}{}_{\bar{X}}} \mathchoice{\hspace{-0.55542pt}}{\hspace{-0.55542pt}}{\hspace{-0.27771pt}}{} (\bar{t})&= \sum _{ a \mathop {\hspace{-1.66656pt}\downharpoonright \hspace{-1.111pt}\mathchoice{\hspace{-1.94443pt}}{\hspace{-1.94443pt}}{}{}_{\bar{X}}} \mathchoice{\hspace{-0.55542pt}}{\hspace{-0.55542pt}}{\hspace{-0.27771pt}}{} = \bar{t}} \partial _{s' \vert t'} q(a), \nonumber \\ \partial _{s' \vert t'} q \mathop {\hspace{-1.66656pt}\downharpoonright \hspace{-1.111pt}\mathchoice{\hspace{-1.94443pt}}{\hspace{-1.94443pt}}{}{}_{\bar{X}}} \mathchoice{\hspace{-0.55542pt}}{\hspace{-0.55542pt}}{\hspace{-0.27771pt}}{} (\bar{t} \rightarrow \bar{s})&= \sum _{ a \mathop {\hspace{-1.66656pt}\downharpoonright \hspace{-1.111pt}\mathchoice{\hspace{-1.94443pt}}{\hspace{-1.94443pt}}{}{}_{\bar{X}}} \mathchoice{\hspace{-0.55542pt}}{\hspace{-0.55542pt}}{\hspace{-0.27771pt}}{} = \bar{t}} \sum _{ d \mathop {\hspace{-1.66656pt}\downharpoonright \hspace{-1.111pt}\mathchoice{\hspace{-1.94443pt}}{\hspace{-1.94443pt}}{}{}_{\bar{X}}} \mathchoice{\hspace{-0.55542pt}}{\hspace{-0.55542pt}}{\hspace{-0.27771pt}}{} = \bar{s}} \partial _{s' \vert t'} q(a \rightarrow _*d). \end{aligned}$$

One building block we need is

$$\begin{aligned}&{\mathcal {D}}_{q}(s' \vert t'; a)\\&:=\partial _{s' \vert t'} q(({t'},\underline{U(s')}) \rightarrow _*a \mid ({t'},\underline{U(s')})) \\&= \sum _{s'':({t'},\underline{U(s')}) \rightarrow s''} \partial _{s' \vert t'} q(s'' \vert ({t'},\underline{U(s')})) q(s'' \rightarrow _*a \mid s'') \\&= \sum _{s'':({t'},\underline{U(s')}) \rightarrow s''} q(s' \vert ({t'},\underline{U(s')})) \left[ 1_{\{s''=s'\}} - q(s'' \vert ({t'},\underline{U(s')})) \right] q(s'' \rightarrow _*a \mid s'') \\&= q(s' \vert ({t'},\underline{U(s')})) \\&\left[ q(s' \rightarrow _*a \mid s') - \sum _{s'':({t'},\underline{U(s')}) \rightarrow s''} q(s'' \vert ({t'},\underline{U(s')})) q(s'' \rightarrow _*a \mid s'') \right] \\&= q(s' \vert ({t'},\underline{U(s')})) \left[ q(s' \rightarrow _*a \mid s') - q(({t'},\underline{U(s')}) \rightarrow _*a \mid ({t'},\underline{U(s')})) \right] . \end{aligned}$$

Note that computing ${\mathcal {D}}_{q}(s' \vert t'; a)$ is a constant time calculation after accumulating a table of path probabilities in linear time before the gradient calculation.

Following an argument similar to Eq. A4, we drop terms that are constant with respect to $s' \vert t'$ and see that,

A8 $$\begin{aligned} \partial _{s' \vert t'} q(a)&= q(t') \partial _{s' \vert t'} q(({t'},\underline{U(s')}) \rightarrow _*a \mid ({t'},\underline{U(s')})) \nonumber \\&= q(t') {\mathcal {D}}_{q}(s' \vert t'; a). \end{aligned}$$

Furthermore, by identical reasoning we calculate the derivative of the path probabilities,

A9 $$\begin{aligned} \partial _{s' \vert t'} q(a \rightarrow _*d)&= \partial _{s' \vert t'} \left[ q(a) q(a \rightarrow _*d \vert a) \right] \nonumber \\&= q(a) \partial _{s' \vert t'} q(a \rightarrow _*d \vert a) + \partial _{s' \vert t'} q(a) q(a \rightarrow _*d \vert a) \nonumber \\&= q(a) q(a \rightarrow _*t' \vert a) \partial _{s' \vert t'} q(({t'},\underline{U(s')}) \rightarrow _*d \mid ({t'},\underline{U(s')})) \nonumber \\&\qquad + q(t') {\mathcal {D}}_{q}(s' \vert t'; a) q(a \rightarrow _*d \vert a) \nonumber \\&= q(a) q(a \rightarrow _*t' \vert a) {\mathcal {D}}_{q}(s' \vert t'; d) \nonumber \\&\qquad + q(t') {\mathcal {D}}_{q}(s' \vert t'; a) q(a \rightarrow _*d \vert a). \end{aligned}$$

We combine Eqs. A6 and A7 to find our KL derivative,

$$\begin{aligned}&\partial _{s' \vert t'} D_{\text {KL}}(p \parallel q \mathop {\hspace{-1.66656pt}\downharpoonright \hspace{-1.111pt}\mathchoice{\hspace{-1.94443pt}}{\hspace{-1.94443pt}}{}{}_{\bar{X}}} \mathchoice{\hspace{-0.55542pt}}{\hspace{-0.55542pt}}{\hspace{-0.27771pt}}{} )\\&= -\sum _{(\bar{t} \rightarrow \bar{s})} \frac{p(\bar{t} \rightarrow \bar{s})}{ q \mathop {\hspace{-1.66656pt}\downharpoonright \hspace{-1.111pt}\mathchoice{\hspace{-1.94443pt}}{\hspace{-1.94443pt}}{}{}_{\bar{X}}} \mathchoice{\hspace{-0.55542pt}}{\hspace{-0.55542pt}}{\hspace{-0.27771pt}}{} (\bar{s} \vert \bar{t})} \, \partial _{s' \vert t'} \, q \mathop {\hspace{-1.66656pt}\downharpoonright \hspace{-1.111pt}\mathchoice{\hspace{-1.94443pt}}{\hspace{-1.94443pt}}{}{}_{\bar{X}}} \mathchoice{\hspace{-0.55542pt}}{\hspace{-0.55542pt}}{\hspace{-0.27771pt}}{} (\bar{s} \vert \bar{t}), \\&= -\sum _{(\bar{t} \rightarrow \bar{s})} \frac{p(\bar{t} \rightarrow \bar{s})}{ q \mathop {\hspace{-1.66656pt}\downharpoonright \hspace{-1.111pt}\mathchoice{\hspace{-1.94443pt}}{\hspace{-1.94443pt}}{}{}_{\bar{X}}} \mathchoice{\hspace{-0.55542pt}}{\hspace{-0.55542pt}}{\hspace{-0.27771pt}}{} (\bar{s} \vert \bar{t})} \, \frac{1}{ q \mathop {\hspace{-1.66656pt}\downharpoonright \hspace{-1.111pt}\mathchoice{\hspace{-1.94443pt}}{\hspace{-1.94443pt}}{}{}_{\bar{X}}} \mathchoice{\hspace{-0.55542pt}}{\hspace{-0.55542pt}}{\hspace{-0.27771pt}}{} (\bar{t})} \left[ \partial _{s' \vert t'} q \mathop {\hspace{-1.66656pt}\downharpoonright \hspace{-1.111pt}\mathchoice{\hspace{-1.94443pt}}{\hspace{-1.94443pt}}{}{}_{\bar{X}}} \mathchoice{\hspace{-0.55542pt}}{\hspace{-0.55542pt}}{\hspace{-0.27771pt}}{} (\bar{t} \rightarrow \bar{s}) - q \mathop {\hspace{-1.66656pt}\downharpoonright \hspace{-1.111pt}\mathchoice{\hspace{-1.94443pt}}{\hspace{-1.94443pt}}{}{}_{\bar{X}}} \mathchoice{\hspace{-0.55542pt}}{\hspace{-0.55542pt}}{\hspace{-0.27771pt}}{} (\bar{s} \vert \bar{t}) \cdot \partial _{s' \vert t'} q \mathop {\hspace{-1.66656pt}\downharpoonright \hspace{-1.111pt}\mathchoice{\hspace{-1.94443pt}}{\hspace{-1.94443pt}}{}{}_{\bar{X}}} \mathchoice{\hspace{-0.55542pt}}{\hspace{-0.55542pt}}{\hspace{-0.27771pt}}{} (\bar{t}) \right] \\&= \sum _{(\bar{t} \rightarrow \bar{s})} \frac{p(\bar{t})}{ q \mathop {\hspace{-1.66656pt}\downharpoonright \hspace{-1.111pt}\mathchoice{\hspace{-1.94443pt}}{\hspace{-1.94443pt}}{}{}_{\bar{X}}} \mathchoice{\hspace{-0.55542pt}}{\hspace{-0.55542pt}}{\hspace{-0.27771pt}}{} (\bar{t})} \frac{p(\bar{s} \vert \bar{t})}{ q \mathop {\hspace{-1.66656pt}\downharpoonright \hspace{-1.111pt}\mathchoice{\hspace{-1.94443pt}}{\hspace{-1.94443pt}}{}{}_{\bar{X}}} \mathchoice{\hspace{-0.55542pt}}{\hspace{-0.55542pt}}{\hspace{-0.27771pt}}{} (\bar{s} \vert \bar{t})} \left[ q \mathop {\hspace{-1.66656pt}\downharpoonright \hspace{-1.111pt}\mathchoice{\hspace{-1.94443pt}}{\hspace{-1.94443pt}}{}{}_{\bar{X}}} \mathchoice{\hspace{-0.55542pt}}{\hspace{-0.55542pt}}{\hspace{-0.27771pt}}{} (\bar{s} \vert \bar{t}) \cdot \partial _{s' \vert t'} q \mathop {\hspace{-1.66656pt}\downharpoonright \hspace{-1.111pt}\mathchoice{\hspace{-1.94443pt}}{\hspace{-1.94443pt}}{}{}_{\bar{X}}} \mathchoice{\hspace{-0.55542pt}}{\hspace{-0.55542pt}}{\hspace{-0.27771pt}}{} (\bar{t}) - \partial _{s' \vert t'} q \mathop {\hspace{-1.66656pt}\downharpoonright \hspace{-1.111pt}\mathchoice{\hspace{-1.94443pt}}{\hspace{-1.94443pt}}{}{}_{\bar{X}}} \mathchoice{\hspace{-0.55542pt}}{\hspace{-0.55542pt}}{\hspace{-0.27771pt}}{} (\bar{t} \rightarrow \bar{s}) \right] \\&= \sum _{\bar{t}} \frac{p(\bar{t})}{ q \mathop {\hspace{-1.66656pt}\downharpoonright \hspace{-1.111pt}\mathchoice{\hspace{-1.94443pt}}{\hspace{-1.94443pt}}{}{}_{\bar{X}}} \mathchoice{\hspace{-0.55542pt}}{\hspace{-0.55542pt}}{\hspace{-0.27771pt}}{} (\bar{t})} \partial _{s' \vert t'} q \mathop {\hspace{-1.66656pt}\downharpoonright \hspace{-1.111pt}\mathchoice{\hspace{-1.94443pt}}{\hspace{-1.94443pt}}{}{}_{\bar{X}}} \mathchoice{\hspace{-0.55542pt}}{\hspace{-0.55542pt}}{\hspace{-0.27771pt}}{} (\bar{t}) \sum _{\bar{s}:\bar{t} \rightarrow \bar{s}} p(\bar{s} \vert \bar{t}) \\&\quad -\sum _{(\bar{t} \rightarrow \bar{s})} \frac{p(\bar{t})}{ q \mathop {\hspace{-1.66656pt}\downharpoonright \hspace{-1.111pt}\mathchoice{\hspace{-1.94443pt}}{\hspace{-1.94443pt}}{}{}_{\bar{X}}} \mathchoice{\hspace{-0.55542pt}}{\hspace{-0.55542pt}}{\hspace{-0.27771pt}}{} (\bar{t})} \frac{p(\bar{s} \vert \bar{t})}{ q \mathop {\hspace{-1.66656pt}\downharpoonright \hspace{-1.111pt}\mathchoice{\hspace{-1.94443pt}}{\hspace{-1.94443pt}}{}{}_{\bar{X}}} \mathchoice{\hspace{-0.55542pt}}{\hspace{-0.55542pt}}{\hspace{-0.27771pt}}{} (\bar{s} \vert \bar{t})} \partial _{s' \vert t'} q \mathop {\hspace{-1.66656pt}\downharpoonright \hspace{-1.111pt}\mathchoice{\hspace{-1.94443pt}}{\hspace{-1.94443pt}}{}{}_{\bar{X}}} \mathchoice{\hspace{-0.55542pt}}{\hspace{-0.55542pt}}{\hspace{-0.27771pt}}{} (\bar{t} \rightarrow \bar{s}) \\&= \sum _{\bar{t}} k_{\bar{t}} \frac{p(\bar{t})}{ q \mathop {\hspace{-1.66656pt}\downharpoonright \hspace{-1.111pt}\mathchoice{\hspace{-1.94443pt}}{\hspace{-1.94443pt}}{}{}_{\bar{X}}} \mathchoice{\hspace{-0.55542pt}}{\hspace{-0.55542pt}}{\hspace{-0.27771pt}}{} (\bar{t})} \partial _{s' \vert t'} q \mathop {\hspace{-1.66656pt}\downharpoonright \hspace{-1.111pt}\mathchoice{\hspace{-1.94443pt}}{\hspace{-1.94443pt}}{}{}_{\bar{X}}} \mathchoice{\hspace{-0.55542pt}}{\hspace{-0.55542pt}}{\hspace{-0.27771pt}}{} (\bar{t}) \\&\quad -\sum _{(\bar{t} \rightarrow \bar{s})} \frac{p(\bar{t})}{ q \mathop {\hspace{-1.66656pt}\downharpoonright \hspace{-1.111pt}\mathchoice{\hspace{-1.94443pt}}{\hspace{-1.94443pt}}{}{}_{\bar{X}}} \mathchoice{\hspace{-0.55542pt}}{\hspace{-0.55542pt}}{\hspace{-0.27771pt}}{} (\bar{t})} \frac{p(\bar{s} \vert \bar{t})}{ q \mathop {\hspace{-1.66656pt}\downharpoonright \hspace{-1.111pt}\mathchoice{\hspace{-1.94443pt}}{\hspace{-1.94443pt}}{}{}_{\bar{X}}} \mathchoice{\hspace{-0.55542pt}}{\hspace{-0.55542pt}}{\hspace{-0.27771pt}}{} (\bar{s} \vert \bar{t})} \partial _{s' \vert t'} q \mathop {\hspace{-1.66656pt}\downharpoonright \hspace{-1.111pt}\mathchoice{\hspace{-1.94443pt}}{\hspace{-1.94443pt}}{}{}_{\bar{X}}} \mathchoice{\hspace{-0.55542pt}}{\hspace{-0.55542pt}}{\hspace{-0.27771pt}}{} (\bar{t} \rightarrow \bar{s}) \\&= \sum _{\bar{t}} k_{\bar{t}} \frac{p(\bar{t})}{ q \mathop {\hspace{-1.66656pt}\downharpoonright \hspace{-1.111pt}\mathchoice{\hspace{-1.94443pt}}{\hspace{-1.94443pt}}{}{}_{\bar{X}}} \mathchoice{\hspace{-0.55542pt}}{\hspace{-0.55542pt}}{\hspace{-0.27771pt}}{} (\bar{t})} \sum _{ a \mathop {\hspace{-1.66656pt}\downharpoonright \hspace{-1.111pt}\mathchoice{\hspace{-1.94443pt}}{\hspace{-1.94443pt}}{}{}_{\bar{X}}} \mathchoice{\hspace{-0.55542pt}}{\hspace{-0.55542pt}}{\hspace{-0.27771pt}}{} = \bar{t}} \partial _{s' \vert t'} q(a) \\&\quad -\sum _{(\bar{t} \rightarrow \bar{s})} \frac{p(\bar{t})}{ q \mathop {\hspace{-1.66656pt}\downharpoonright \hspace{-1.111pt}\mathchoice{\hspace{-1.94443pt}}{\hspace{-1.94443pt}}{}{}_{\bar{X}}} \mathchoice{\hspace{-0.55542pt}}{\hspace{-0.55542pt}}{\hspace{-0.27771pt}}{} (\bar{t})} \frac{p(\bar{s} \vert \bar{t})}{ q \mathop {\hspace{-1.66656pt}\downharpoonright \hspace{-1.111pt}\mathchoice{\hspace{-1.94443pt}}{\hspace{-1.94443pt}}{}{}_{\bar{X}}} \mathchoice{\hspace{-0.55542pt}}{\hspace{-0.55542pt}}{\hspace{-0.27771pt}}{} (\bar{s} \vert \bar{t})} \sum _{ a \mathop {\hspace{-1.66656pt}\downharpoonright \hspace{-1.111pt}\mathchoice{\hspace{-1.94443pt}}{\hspace{-1.94443pt}}{}{}_{\bar{X}}} \mathchoice{\hspace{-0.55542pt}}{\hspace{-0.55542pt}}{\hspace{-0.27771pt}}{} = \bar{t}} \sum _{ d \mathop {\hspace{-1.66656pt}\downharpoonright \hspace{-1.111pt}\mathchoice{\hspace{-1.94443pt}}{\hspace{-1.94443pt}}{}{}_{\bar{X}}} \mathchoice{\hspace{-0.55542pt}}{\hspace{-0.55542pt}}{\hspace{-0.27771pt}}{} = \bar{s}} \partial _{s' \vert t'} q(a \rightarrow _*d), \end{aligned}$$

where $k_{\bar{t}}$ is the number of child clades of $\bar{t}$ of size 2 or larger, and therefore have a probability distribution of child subsplits to sum over. After the linear pass through the support accumulating path probabilities, the algorithmic efficiency of this calculation is $O(n_{p}\cdot n_{p} \mathop {\hspace{-1.66656pt}\downharpoonright \hspace{-1.111pt}\mathchoice{\hspace{-1.94443pt}}{\hspace{-1.94443pt}}{}{}_{\bar{X}}} \mathchoice{\hspace{-0.55542pt}}{\hspace{-0.55542pt}}{\hspace{-0.27771pt}}{} )$, where $n_{p}$ is the number of PCSPs in the support, and $n_{p} \mathop {\hspace{-1.66656pt}\downharpoonright \hspace{-1.111pt}\mathchoice{\hspace{-1.94443pt}}{\hspace{-1.94443pt}}{}{}_{\bar{X}}} \mathchoice{\hspace{-0.55542pt}}{\hspace{-0.55542pt}}{\hspace{-0.27771pt}}{}$ is the number of paths in the support that restrict to a PCSP on tip set $\bar{X}$.

### Regularization Penalty

Tree space grows superexponentially in the number of tips, so reference distributions with less overlap may not contain enough information to uniquely specify the supertree distribution. To alleviate this, we implemented an optional regularization penalty in our supertree loss function, encouraging probabilistic uncertainty in the SBN conditional distributions in the absence of information from the references.

Experiments revealed that penalizing simply using information entropy had undesirable gradient properties. However, higher-entropy distributions will have probabilities that are relatively even, so we elect to penalize large differences in SBN softmax parameters. We implemented the penalty function

$$\begin{aligned} R(q) = \frac{1}{2}\sum _{t \rightarrow s} (v_{s \vert t} - \bar{v}_{\cdot \vert t})^2 \end{aligned}$$

summing the squared differences between each softmax parameter $v_{s \vert t}$ and the average of all of the softmax parameters in its parent’s conditional distribution $\bar{v}_{\cdot \vert t}$. Differentiation leads to the gradient

$$\begin{aligned} \partial _{s' \vert t'} R(q) = v_{s' \vert t'} - \bar{v}_{\cdot \vert t'} \end{aligned}$$

which encourages conditional probabilities to become more similar when used in gradient descent. We use a regularization weight $\lambda$ multiplied by the penalty function *R*.

Repeating our simulated and real-world data vbsupertree experiments with $\lambda =0$ reproduces our results from Sect. 3. Increasing the penalty to $\lambda =0.05$ leads to similar results for both our simulated data (Fig. 10) and our HCV data (Fig. 11). We see smooth operation of the vbsupertree algorithm, but with slightly higher converged KL divergence versus the truth. This is to be expected, as regularization penalties bias results on training data in return for better performance in other areas. Finally, raising the penalty higher to $\lambda =0.50$ introduces an interesting effect. The KL versus truth plot consistently falls, then rebounds slightly. We intend to explore the effects of different $\lambda$ values in future work adapting machine learning validation techniques to improve training SBNs (Figs. 12, 13).

## Code Availability

A Python implementation of the mutualization and vbsupertree methods can be found at https://github.com/mdkarcher/vbsupertree. (GitHub repo/Python package)
